# Nonlinear association between first-trimester plasma aldosterone concentration and risk of hypertensive disorders of pregnancy: a multicenter prospective cohort study

**DOI:** 10.3389/fendo.2026.1836569

**Published:** 2026-05-25

**Authors:** Nanfang Li, Mei Yang, Jinxuan Ren, Menghui Wang, Qing Zhu, Bingxuan Guo, Ting Wu, Yue Lin

**Affiliations:** 1 Hypertension Center of People’s Hospital of Xinjiang Uygur Autonomous Region, Xinjiang Hypertension Institute, NHC Key Laboratory of Hypertension Clinical Research, Key Laboratory of Xinjiang Uygur Autonomous Region “Hypertension Research Laboratory”, Xinjiang Clinical Medical Research Center for Hypertension (Cardio-Cerebrovascular) Diseases, Urumqi, Xinjiang, China; 2 Xinjiang Medical University, Urumqi, Xinjiang, China

**Keywords:** aldosterone, association, cohort study, first-trimester, hypertensive disorders of pregnancy

## Abstract

**Background:**

Hypertensive disorders of pregnancy (HDP) threaten maternal and fetal health, and the etiology remains unclear. Previous studies have reported lower plasma aldosterone concentration (PAC) in patients with HDP, but this association remains controversial. The primary objective of this study was to determine the relationship between first-trimester PAC and the risk of HDP.

**Methods:**

This prospective, multicenter cohort study recruited eligible pregnant women in their first-trimester from six hospitals in China between May 1, 2023, and December 31, 2024. Inclusion criteria comprised naturally conceived singleton pregnancy with a gestational age of less than 14 weeks. The primary exposure was the baseline PAC measured at first-trimester pregnancy registration. The primary outcome was a clinical diagnosis of HDP. The association was assessed using multivariable logistic regression, adjusting for potential confounders. Potential nonlinearity was explored with restricted cubic splines (RCS), and threshold effects were evaluated using piecewise regression. Sensitivity analyses were conducted to test the robustness of the findings. Statistical analyses were performed using R software (version 4.2.2) and SPSS (version 26.0). A two-sided *P*-value < 0.050 was considered statistically significant.

**Results:**

A total of 1,486 women were included in the final analysis, of whom 145 (9.8%) developed HDP. Women with HDP had significantly lower first-trimester PAC than those without (median: 18.85 vs. 22.96 ng/dL, *P* < 0.001). The RCS analysis revealed a significant nonlinear relationship between PAC and HDP risk (*P* for nonlinearity = 0.005). Piecewise regression identified a threshold at 39.30 ng/dL: below it, each 1 ng/dL increase in PAC was associated with a 3.8% reduction in the odds of HDP (adjusted odds ratio [OR], 0.962, 95% confidence interval [CI]: 0.939–0.985, *P* = 0.001); at or above it, the association was positive but not significant (adjusted OR, 1.027, 95% CI: 0.995–1.060, *P* = 0.098). These findings remained consistent across multiple sensitivity analyses.

**Conclusion:**

First-trimester PAC exhibited a nonlinear association with HDP risk, with an inflection point at 39.30 ng/dL. Below this threshold, higher PAC was significantly associated with a lower risk of HDP. At or above this level, the association was positive but not statistically significant.

## 1 Introduction

Hypertensive disorders of pregnancy (HDP) represent common obstetric complications that are strongly associated with severe pregnancy outcomes (1–5). Globally, HDP affect approximately 6–10% of pregnancies and account for nearly 76,000 maternal deaths and 500,000 fetal and newborn deaths each year (6). As one of the leading causes of maternal and fetal morbidity and mortality worldwide (7), HDP remain a major public health concern. Despite extensive research, their pathogenesis has not been fully elucidated (8). Identifying early, measurable risk factors is therefore crucial for developing effective prevention strategies to improve maternal and fetal outcomes.

HDP comprise a spectrum of conditions represented by preeclampsia. Extensive research has identified multiple etiological factors contributing to their development, including impaired uteroplacental perfusion and oxygenation, heightened inflammatory responses, immune dysregulation, and imbalances in angiogenic factors. These pathophysiological mechanisms collectively contribute to the development and progression of HDP (9). Recent studies have shown that the renin-angiotensin-aldosterone system (RAAS) in the peripheral blood of patients with HDP exhibits regulatory abnormalities (10). As a key end product of RAAS, plasma aldosterone concentration (PAC) has been found to be lower in women with HDP, yet their association remains controversial. Current literature lacks definitive longitudinal evidence to clarify whether the relationship between first-trimester PAC and HDP reflects a potential causal link or merely a non-causal correlation. Therefore, prospective cohort studies are urgently needed to fill this knowledge gap.

Given the hypothesis of a nonlinear association between PAC and HDP risk, a prospective, multicenter cohort study was designed to investigate this relationship. The study aims to provide new insights into the pathogenesis of HDP and identify potential early biomarkers, which may inform future preventive and therapeutic strategies.

## 2 Materials and methods

### 2.1 Study design, participants, and ethical approval

This prospective, multicenter cohort study consecutively enrolled eligible participants during their first-trimester of pregnancy.

Participants were recruited from six hospitals in Xinjiang Uygur Autonomous Region, China, between May 1, 2023, and December 31, 2024: the People’s Hospital of Xinjiang Uygur Autonomous Region, the People’s Hospital of Tacheng Region, the People’s Hospital of Emin County, the People’s Hospital of Wusu City, the People’s Hospital of Tuoli County, and the People’s Hospital of the Ninth Division.

#### 2.1.1 Inclusion and exclusion criteria

**Figure 1:**
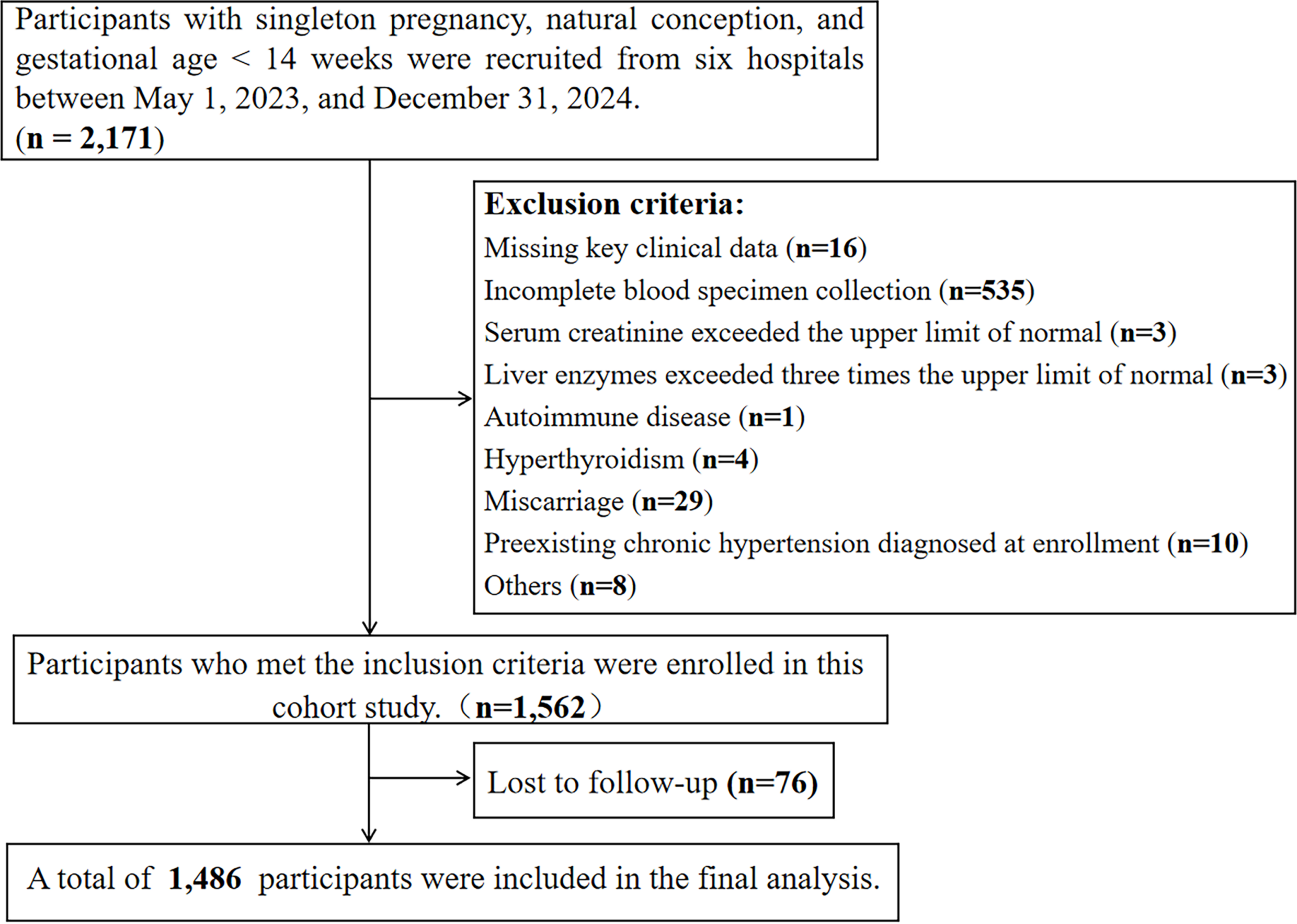
Study flowchart: enrollment, exclusions, and follow-up of the prospective pregnancy cohort. A total of 2,171 women in the first trimester of pregnancy were screened for eligibility across six collaborating hospitals between May 2023 and December 2024. After applying the exclusion criteria, 1,562 women were enrolled. During follow-up, 76 participants were lost to follow-up, resulting in a final analytical cohort of 1,486 participants with complete first-trimester PAC measurements and outcome data for the primary analysis of PAC and HDP risk.

To establish a well-defined cohort and minimize confounding, eligible participants were required to meet all of the following inclusion criteria and none of the exclusion criteria. Participants were included if they: (1) Had a naturally conceived, singleton pregnancy confirmed by ultrasonography. (2) Were enrolled at a gestational age of <14 weeks, with pregnancy registration established at a collaborating hospital and the intention to receive all antenatal care and deliver at one of collaborating hospitals. (3) Were capable of providing written informed consent, complying with the follow-up schedule, and authorizing access to clinical data, with no known psychiatric or cognitive disorders. Participants were excluded for any of the following: (1) Preexisting chronic hypertension diagnosed at the time of enrollment. (2) Abnormal liver or kidney function, defined as alanine aminotransferase (ALT) or aspartate aminotransferase (AST) >3 times the upper limit of normal, or elevated serum creatinine. (3) A history of autoimmune diseases, renal disorders known to affect aldosterone metabolism, hyperthyroidism, Addison’s disease, Cushing’s syndrome, or other endocrine disorders that alter aldosterone synthesis or metabolism. (4) Use of medications known to influence PAC (e.g., mineralocorticoid receptor antagonists, angiotensin-converting enzyme inhibitors, or glucocorticoids) within the month preceding enrollment. (5) Concurrent pregnancy-related complications (e.g., intrahepatic cholestasis of pregnancy), pregnancy loss, or planned termination of pregnancy. (6) Missing key clinical data, anticipated inadequate follow-up compliance, or an inadequate blood sample for PAC measurement. The participant selection process is summarized in **Figure 1**.

#### 2.1.2 Participant follow-up

At enrollment (first-trimester pregnancy registration), baseline data and blood samples were collected. All participants were then prospectively followed from enrollment until 12 weeks postpartum through routine clinical visits and structured telephone interviews. Details regarding the follow-up schedule, definitions, and management of loss to follow-up are provided in the **Supplementary Material**.

#### 2.1.3 Ethical approval

Ethical approval was obtained from the Ethics Committee of the People’s Hospital of Xinjiang Uygur Autonomous Region (Approval No: KY2022120102; Date: December 1, 2022). All participants provided written informed consent. The study was conducted in accordance with the principles of the Declaration of Helsinki.

### 2.2 Baseline data collection

Trained research nurses collected baseline information through face-to-face interviews and review of medical records using standardized forms. Collected information included: (1) Clinical characteristics: age, body mass index (BMI), gravidity, parity, blood pressure, hemoglobin, leukocyte and platelet counts, ALT, AST, albumin, fasting glucose, serum creatinine, blood urea nitrogen, calcium levels, and gestational age (determined by last menstrual period and ultrasound confirmation). (2) Medical history: miscarriage, stillbirth, HDP, and diabetes. (3) Family history: parental hypertension. All data were entered into a secure, encrypted electronic database within 24 hours.

### 2.3 PAC measurement

Blood collection and processing: At study enrollment, fasting venous blood was collected from the antecubital vein of all participants between 8:00 and 10:00 AM local time, following a 30-minute seated rest. Blood was drawn into EDTA tubes and centrifuged (3000×g, 15 min, room temperature) to obtain plasma. Plasma aliquots were stored at –20 °C and transferred within one month on dry ice to the laboratory of the Hypertension Institute, where they were stored at –80 °C until batch analysis. The laboratory specializes in endocrine hormone measurement and maintains a dedicated team and internal quality control procedures for such assays.

Aldosterone assay: PAC was quantified in a single batch at the laboratory of the Hypertension Institute using a chemiluminescent microparticle immunoassay technology on an AutoLumo A2000 Plus analyzer (Autobio Diagnostics, Zhengzhou, China), strictly following the manufacturer’s instructions. Assay precision was validated using low- and high-concentration quality controls. Intra-assay precision was assessed from 25 replicate measurements within a single run, and inter-assay precision was determined across five consecutive batches. The intra- and inter-assay coefficients of variation were 5.81% and 8.10% for the low control, and 3.33% and 7.65% for the high control, respectively.

### 2.4 Outcome ascertainment

The primary outcome was the development of HDP, including gestational hypertension, preeclampsia, eclampsia, and chronic hypertension with or without superimposed preeclampsia (11), as defined by the American College of Obstetricians and Gynecologists (ACOG) guidelines (12).

Outcome data were extracted from antenatal, delivery, and postpartum medical records. To ensure blinded adjudication, two attending obstetricians independently reviewed all cases against the ACOG criteria. Any discrepancy was resolved by a third senior obstetrician, whose decision was final.

### 2.5 Statistical analysis

Continuous variables were presented as median (interquartile range) and categorical variables as number (percentage). As continuous variables were not normally distributed, group comparisons were performed using the Mann-Whitney U test or Kruskal-Wallis test for continuous variables and the chi-square or Fisher’s exact test for categorical variables, as appropriate. Missing data, complete blood count (n=7) and serum calcium (n=4), accounted for < 2% of the total dataset and were imputed using the mean.

The association between PAC and HDP was analyzed using multivariable logistic regression. Thecovariates for the primary model were selected *a priori*, based on clinical relevance and a pre-specified causal framework informed by previous literature (12–15). To assess the robustness of this pre-specified model, an exploratory analysis was performed using a backward stepwise selection procedure (**Supplementary Tables 1**, **2**). To account for inter-center variation, PAC values were standardized (Z-score) within each center. Results were expressed as odds ratios (OR) with 95% confidence intervals (CIs) (16).

A potential nonlinear relationship was explored using restricted cubic splines (RCS), and a threshold effect was examined with piecewise regression. Sensitivity analyses were conducted to examine the robustness of the findings.

All statistical analyses were performed using R software (version 4.2.2) and SPSS (version 26.0). A two-sided *P* value < 0.050 was considered statistically significant.

### 2.6 Quality control

A comprehensive quality control system was implemented throughout the study to ensure scientific rigor and data reliability. It included standardized protocols for personnel training, procedural supervision, and data management. Detailed descriptions are provided in the **Supplementary Material**.

## 3 Results

### 3.1 Baseline characteristics of the study population

A total of 1,486 eligible pregnant women with first-trimester PAC measurement were included. Baseline characteristics were as follows: a median age of 29 years (IQR, 27–33), a median BMI of 22.67 kg/m²(IQR, 20.54–25.39), a median gestational age of 8 weeks (IQR, 6–11), and a median PAC of 22.32 ng/dL (IQR, 15.59–33.40).

Among these women, 145 (9.8%) were diagnosed with HDP: 68 had gestational hypertension, 68 had preeclampsia, and 9 had chronic hypertension (defined as *de novo*hypertension during pregnancy that persisted beyond 12 weeks postpartum). When participants were stratified by PAC quartiles (Q1: <15.59 ng/dL; Q2: 15.59–22.31 ng/dL; Q3: 22.31–33.40 ng/dL; Q4: ≥33.40 ng/dL), several baseline characteristics, including age, BMI, hemoglobin, serum creatinine, blood urea nitrogen, and albumin levels, differed significantly (all *P* < 0.050, **Supplementary Table 3**). With increasing PAC quartiles, diastolic blood pressure (DBP) decreased (*P* = 0.017), plasma renin concentration increased (*P* < 0.001), and the prevalence of HDP exhibited a reduction (*P* = 0.004).

### 3.2 Association between PAC and HDP risk

When analyzed as a continuous variable, each 1 ng/dL increase in first-trimester PAC was associated with lower odds of HDP in the unadjusted model (OR, 0.986; 95% CI, 0.973–0.996; *P* = 0.014). This inverse association remained statistically significant after sequential adjustment for maternal age, BMI, history of HDP, gestational age, and baseline blood pressure (all *P* < 0.050). Similarly, per standard deviation increase in standardized PAC was inversely associated with HDP risk in both unadjusted and adjusted models (all *P* < 0.050; **Table 1**).

**Table 1:** Association between PAC and HDP risk.

Characteristics	Model 1			Model 2			Model 3		
	OR	95% CI	*P*-value	OR	95% CI	*P*-value	OR	95% CI	*P*-value
PAC	0.986	0.973, 0.996	0.014	0.988	0.975, 0.999	0.037	0.987	0.974, 0.999	0.039
PAC^*^	0.762	0.606, 0.935	0.014	0.791	0.627, 0.974	0.037	0.784	0.614, 0.975	0.039
PAC group									
Q1 < 15.59	Ref.	Ref.		Ref.	Ref.		Ref.	Ref.	
Q2: 15.59-22.31	0.884	0.571, 1.366	0.579	0.811	0.517, 1.268	0.359	0.804	0.500, 1.288	0.366
Q3: 22.31-33.40	0.492	0.295, 0.804	0.005	0.473	0.281, 0.781	0.004	0.478	0.278, 0.806	0.006
Q4 ≥ 33.40	0.495	0.297, 0.809	0.006	0.518	0.308, 0.853	0.011	0.507	0.295, 0.857	0.012
*P* for trend			<0.001			0.002			0.002

Model 1, no covariates were adjusted.

Model 2, adjusted for age, BMI, and history of HDP.

Model 3, adjusted for age, BMI, history of HDP, gestational age, baseline SBP and DBP.

^*^ standardized PAC.

BMI, Body mass index; DBP, Diastolic blood pressure; HDP, Hypertensive disorders of pregnancy; SBP, Systolic blood pressure; PAC, Plasma aldosterone concentration; OR, Odds Ratio; CI, Confidence Interval.

When analyzed by quartiles, an inverse dose-response relationship was observed in both unadjusted (*P* for trend <0.001) and adjusted models (*P* for trend = 0.002). Compared with Q1 (PAC < 15.59 ng/dL), only Q3 (22.31–33.40 ng/dL) and Q4 (≥33.40 ng/dL) showed significantly lower odds of HDP (**Table 1**).

### 3.3 Association analysis after winsorization of PAC

#### 3.3.1 PAC and mitigation of extreme value effects

To minimize the influence of extreme values, we winsorized PAC at the 5th and 95th percentiles, reducing the range from 6.95–125.01 ng/dL to 10.22–68.08 ng/dL. In analyses using winsorized PAC, women with HDP had significantly lower median PAC levels than those without HDP [18.85 *(IQR 13.70–28.23) vs. 22.96 ng/dL (IQR 15.87–33.72); *P* < 0.001]. Moreover, the prevalence of HDP decreased across ascending PAC quartiles: 13.2% in Q1 (<15.59 ng/dL), 11.9% in Q2 (15.59–22.31 ng/dL), 7.0% in Q3 (22.31–33.40 ng/dL), and 7.0% in Q4 (≥33.40 ng/dL) (*P* for trend = 0.004).

#### 3.3.2 Association between winsorized PAC and HDP risk

After winsorizing PAC, an inverse association between PAC and HDP risk persisted across all analytical models. In continuous analysis, each 1 ng/dL increase in winsorized PAC was associated with lower HDP odds in the unadjusted model (OR, 0.984, 95% CI 0.971–0.997; *P* = 0.014), and this remained significant in multivariable-adjusted models (all *P* < 0.050). Similarly, per standard deviation increase in standardized PAC was inversely associated with HDP risk (OR, 0.781, 95% CI 0.641–0.951; *P* = 0.014), with consistent significance upon adjustment (all *P* < 0.050; **Supplementary Table 4**).

In categorical analysis, an inverse dose-response relationship was observed across PAC quartiles in both unadjusted (*P* for trend <0.001) and adjusted models (*P* for trend = 0.002). Compared with Q1 (PAC < 15.59 ng/dL), significantly lower odds of HDP were observed in Q3 (22.31–33.40 ng/dL) and Q4 (≥33.40 ng/dL) (**Supplementary Table 4**).

### 3.4 Nonlinear association between winsorized PAC and HDP risk

To characterize the potential nonlinear association, we performed restricted cubic spline (RCS) regression with knots placed at the 10th, 50th, and 90th percentiles of the PAC distribution (selected by minimizing the Akaike Information Criterion), adjusting for maternal age, BMI, HDP history, gestational age, baseline systolic blood pressure (SBP), and baseline DBP.

**Figure 2:**
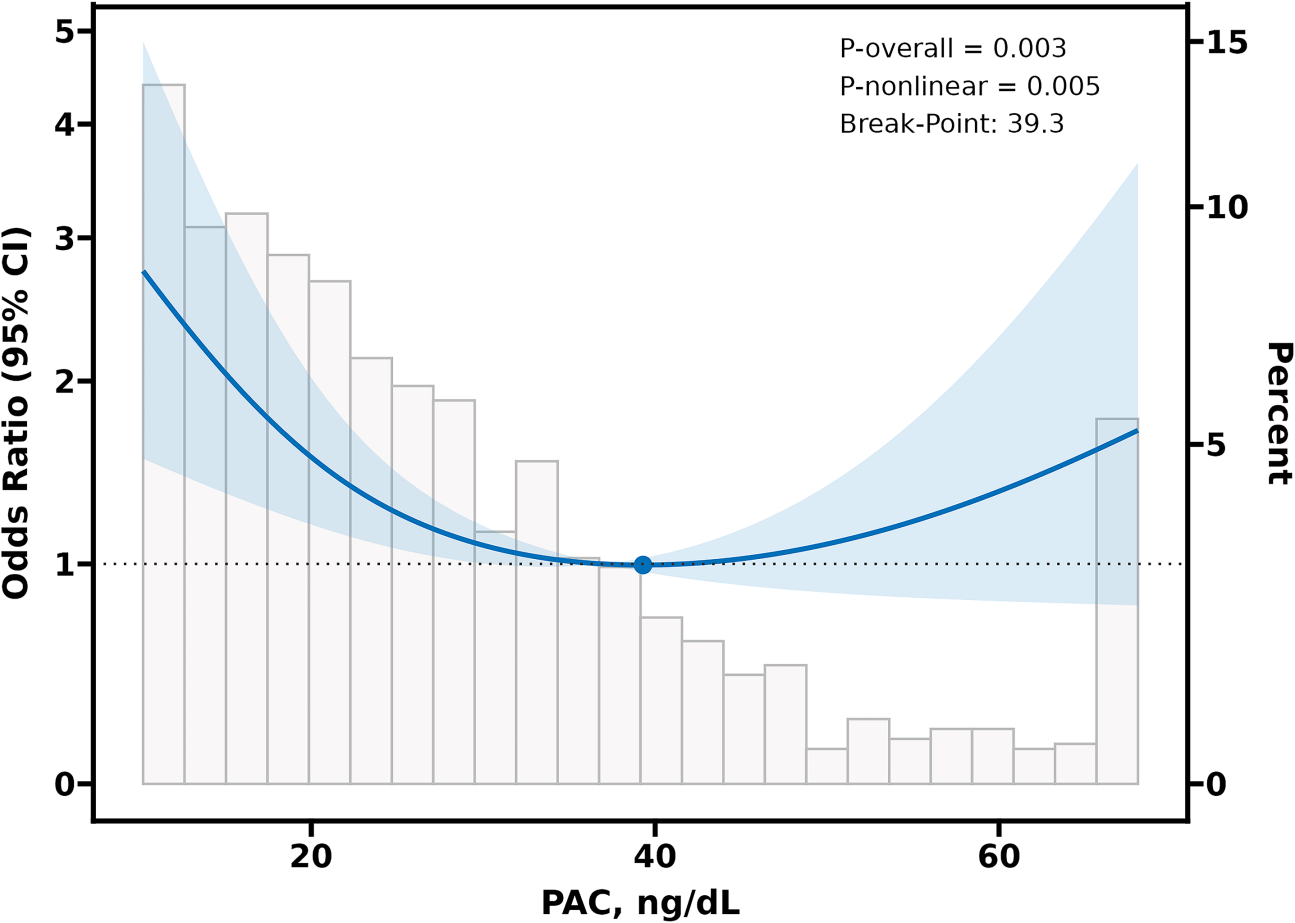
Nonlinear association between winsorized PAC and HDP risk. RCS analysis depicting the dose-response relationship between winsorized PAC (x-axis) and the adjusted OR (y-axis) for HDP. The solid blue curve represents the estimated OR, and the shaded blue area indicates the 95% confidence interval. A significant nonlinear association was observed (*P* for nonlinearity = 0.005; *P* for overall association = 0.003). The inflection point was identified at 39.30 ng/dL. The model was adjusted for maternal age, BMI, history of HDP, gestational age, baseline SBP, and baseline DBP. BMI, Body mass index; DBP, Diastolic blood pressure; HDP, Hypertensive disorders of pregnancy; PAC, Plasma aldosterone concentration; RCS, Restricted cubic splines; SBP, Systolic blood pressure; OR, Odds Ratio.

The RCS analysis revealed a statistically significant overall association between winsorized PAC and HDP risk (*P* for overall = 0.003) with evidence of significant nonlinearity (*P* for nonlinearity = 0.005), indicating that the relationship was not linear across the range of PAC (**Figure 2**).

### 3.5 Threshold analysis and piecewise regression

To quantify the nonlinear association between winsorized PAC and HDP risk, a piecewise regression model was constructed. The knot was set at 39.30 ng/dL, corresponding to the nadir of the preceding RCS curve. This model was adjusted for maternal age, BMI, history of HDP, gestational age, and baseline blood pressure.

Piecewise regression identified a statistically significant threshold effect of PAC on HDP risk (*P* for log-likelihood ratio test = 0.009). Below the threshold of 39.30 ng/dL, each 1 ng/dL increase in PAC was associated with a 3.8% reduction in HDP odds (adjusted OR, 0.962, 95% CI: 0.939–0.985, *P* = 0.001). Conversely, above this threshold, the association was positive but not statistically significant (adjusted OR, 1.027, 95% CI: 0.995–1.060, *P* = 0.098) (**Table 2**).

**Table 2:** Threshold effect analysis of winsorized PAC on HDP risk.

Characteristics	OR (95% CI)^*^	*P*-value
Fitting by standard Logistic regression model	0.986 (0.972, 0.999)	0.044
Fitting by piecewise Logistic regression model (break-points = 39.30 ng/dL)		
PAC < 39.30	0.962 (0.939, 0.985)	0.001
PAC ≥ 39.30	1.027 (0.995, 1.060)	0.098
Log likelihood ratio		0.009

^*^ Adjusted for, age, BMI, history of HDP, gestational age, baseline SBP and DBP.

BMI, Body mass index; DBP, Diastolic blood pressure; HDP, Hypertensive disorders of pregnancy; SBP, Systolic blood pressure; PAC, Plasma aldosterone concentration; OR, Odds Ratio; CI, Confidence Interval.

### 3.6 Sensitivity analyses

#### 3.6.1 Adjustment for recruiting center

To evaluate the influence of inter-center heterogeneity, we repeated all analyses with adjustment for the recruiting center.

The inverse association between first-trimester PAC and HDP risk remained observed. in fully adjusted models, an inverse dose-response relationship was observed across PAC quartiles, with significantly lower odds of HDP in Q3 (22.31–33.40 ng/dL) and Q4 (≥ 33.40 ng/dL) compared with Q1 (< 15.59 ng/dL). These findings were consistent for both raw and winsorized PAC data (**Supplementary Tables 5**, **6**).

The RCS analysis, additionally adjusted for recruiting center, confirmed a significant nonlinear association between winsorized PAC and HDP risk (*P* for overall association = 0.003; *P* for nonlinearity = 0.009), with an inflection point at 40.20 ng/dL (**Supplementary Figure 1**). Subsequent piecewise regression analysis demonstrated a statistically significant threshold effect (*P* for log-likelihood ratio test = 0.011). Below this threshold, PAC was inversely associated with HDP risk (adjusted OR, 0.953; 95% CI, 0.926–0.980; *P* < 0.001), whereas above it, a non-significant positive association was observed (adjusted OR, 1.022; 95% CI, 0.988–1.058; *P* = 0.209) (**Supplementary Table 7**).

#### 3.6.2 Complete-case analysis

To evaluate the potential influence of mean imputation, we conducted a complete-case analysis. The inverse association between first-trimester PAC and HDP risk persisted in all models (all *P* < 0.050). Consistently, an inverse dose-response relationship was observed across PAC quartiles, with Q3 (22.31–33.40 ng/dL) and Q4 (≥ 33.40 ng/dL) showing significantly lower odds of HDP compared with Q1 (< 15.59 ng/dL) in all models. These patterns were consistent for both raw and winsorized PAC data (**Supplementary Tables 8**, **9**).

RCS analysis, adjusted for maternal age, BMI, HDP history, gestational age, and baseline blood pressure, confirmed a significant nonlinear association for winsorized PAC (*P* for overall association = 0.001; *P* for nonlinearity = 0.007), with an inflection point at 37.50 ng/dL (**Supplementary Figure 2**). Subsequent piecewise regression confirmed a significant threshold effect (*P* for log-likelihood ratio test = 0.006). PAC levels below this threshold were inversely associated with HDP risk (adjusted OR, 0.959; 95% CI, 0.936–0.983; *P* < 0.001), whereas levels at or above it showed a non-significant positive association (adjusted OR, 1.026; 95% CI, 0.996–1.057; *P* = 0.087) (**Supplementary Table 10**).

#### 3.6.3 Exclusion of participants with postpartum-diagnosed chronic hypertension

To assess the robustness of our findings to the inclusion of participants with chronic hypertension diagnosed postpartum (n=9), we conducted a sensitivity analysis after their exclusion.

The inverse association between first-trimester PAC and HDP risk remained observed after adjustment for age, BMI, history of HDP, gestational age, and baseline blood pressure (all *P* < 0.050). An inverse dose-response relationship across PAC quartiles was observed, with significantly lower odds of HDP in Q3 (22.31–33.40 ng/dL) and Q4 (≥ 33.40 ng/dL) compared to Q1 (< 15.59 ng/dL). These patterns were consistent for both raw and winsorized PAC data (**Supplementary Tables 11**, **12**).

RCS analysis, conducted in this excluded cohort and adjusted for the same covariates, confirmed a significant nonlinear association for winsorized PAC (*P* for overall association = 0.002; *P* for nonlinearity = 0.004), with an inflection point at 39.00 ng/dL (**Supplementary Figure 3**). Subsequent piecewise regression confirmed a significant threshold effect (*P* for log-likelihood ratio test = 0.006). PAC levels below this threshold were inversely associated with HDP risk (adjusted OR, 0.960; 95% CI, 0.937–0.983; *P* < 0.001), whereas levels at or above it showed a non-significant positive association (adjusted OR, 1.029; 95% CI, 0.997–1.062; P = 0.074) (**Supplementary Table 13**).

#### 3.6.4 Adjustment for baseline blood pressure

To assess the PAC-HDP association independent of baseline blood pressure and to avoid potential overadjustment, we repeated the analyses without adjusting for baseline SBP and DBP.

The inverse association between first-trimester PAC and HDP risk persisted in models that omitted baseline blood pressure (*P* < 0.050). Similarly, an inverse dose-response relationship across PAC quartiles was observed, with Q3 (22.31–33.40 ng/dL) and Q4 (≥33.40 ng/dL) showing significantly lower odds of HDP compared with Q1 (<15.59 ng/dL). These findings were consistent for both raw and winsorized PAC data (**Supplementary Tables 14**, **15**).

RCS analysis, adjusted only for age, BMI, HDP history, and gestational age, confirmed a significant nonlinear association for winsorized PAC (*P* for overall association = 0.003; *P* for nonlinearity = 0.005), with an inflection point at 39.00 ng/dL (**Supplementary Figure 4**). Subsequent piecewise regression confirmed a significant threshold effect (*P* for log-likelihood ratio test = 0.008). Specifically, winsorized PAC levels below this threshold were inversely associated with HDP risk (adjusted OR, 0.963; 95% CI, 0.941–0.985; *P* < 0.001), whereas levels at or above it showed a non-significant positive association (adjusted OR, 1.026; 95% CI, 0.996–1.058; *P* = 0.087) (**Supplementary Table 16**).

#### 3.6.5 Subgroup analysis by plasma renin concentration

To explore the potential influence of plasma renin concentration on the PAC-HDP association, we performed stratified, RCS, and piecewise regression analyses.

When stratified by the median plasma renin concentration (100.58 pg/mL), the association differed between subgroups. In lower levels (< 100.58 pg/mL), the association between PAC and HDP was not statistically significant (OR, 0.990; 95% CI: 0.970–1.010; *P* = 0.460). Conversely, in the higher levels subgroup (≥ 100.58 pg/mL), a significant inverse association was observed (OR, 0.980; 95% CI: 0.960–0.999; *P* = 0.015). No significant interaction was detected between plasma renin concentration and PAC on HDP risk (*P* for interaction = 0.286, **Supplementary Table 17**).

RCS analysis, additionally adjusted for plasma renin concentration, confirmed a significant nonlinear association between winsorized PAC and HDP risk (*P* for overall association = 0.004; *P* for nonlinearity = 0.006), with an inflection point at 39.30 ng/dL (**Supplementary Figure 5**). Subsequent piecewise regression confirmed a significant threshold effect (*P* for log-likelihood ratio test = 0.010). PAC levels below this threshold were inversely associated with HDP risk (adjusted OR, 0.961; 95% CI, 0.938–0.986; *P* < 0.001), whereas levels at or above it showed a non-significant positive association (adjusted OR, 1.027; 95% CI, 0.995–1.060; *P* = 0.098) (**Supplementary Table 18**).

#### 3.6.6 Bootstrap internal validation

To internally validate the stability of the nonlinear association and the estimated inflection point, we performed a bootstrap internal validation with 1,000 resampling repetitions. The inverse association between first-trimester PAC and HDP risk persisted after bootstrap internal validation (*P* < 0.050) (**Supplementary Table 19**). RCS analysis after bootstrap internal validation confirmed a significant nonlinear association between winsorized PAC and HDP risk (*P* for overall association = 0.004; *P* for nonlinearity = 0.006), with an inflection point at 38.20 ng/dL (**Supplementary Figure 6**). Subsequent piecewise regression confirmed a significant threshold effect (*P* for log-likelihood ratio test = 0.008). Winsorized PAC levels below this threshold were inversely associated with HDP risk (adjusted OR, 0.960; 95% CI, 0.942–0.983; *P* = 0.001), whereas levels at or above it showed a non-significant positive association (adjusted OR, 1.029; 95% CI, 0.990–1.062; *P* = 0.104) (**Supplementary Table 20**).

## 4 Discussion

### 4.1 Key findings

This prospective, multicenter cohort study demonstrates that women who subsequently developed HDP had a significantly lower median first-trimester PAC than those who did not. In an initial multivariable logistic regression model, PAC showed an overall inverse association with HDP risk. However, RCS regression revealed a significant nonlinear relationship. Specifically, the association was characterized by a threshold-dependent pattern, with an inflection point estimated at 39.30 ng/dL. Below this threshold, each 1 ng/dL increase in PAC was associated with lower odds of HDP. Conversely, at or above this threshold, the association was positive but not statistically significant. Sensitivity analyses confirmed the robustness of the nonlinear association and the threshold effect, the estimated inflection point remained consistent, ranging narrowly from 37.50 to 40.20 ng/dL across analyses. The sensitivity analyses included the following: (1) adjustment for recruiting center, (2) complete-case analysis (excluding cases with missing data imputation), (3) exclusion of participants with chronic hypertension diagnosed postpartum, (4) analysis without adjustment for baseline blood pressure, (5) subgroup analysis by plasma renin concentration, and (6) analysis after bootstrap internal validation.

This study employed a composite HDP endpoint, consisting of gestational hypertension, preeclampsia, and chronic hypertension cases that first manifested during pregnancy and persisted >12 weeks postpartum. This approach was based on two key considerations. First, gestational hypertension and preeclampsia represent a clinical and pathophysiological continuum, sharing common mechanistic pathways. And all included chronic hypertension cases had onset during gestation. Second, subtype specific sample sizes were limited, precluding adequately powered stratified analyses. The composite endpoint enhanced statistical efficiency and analytical robustness. Additionally, to confirm that the inclusion of postpartum-persistent cases did not influence the conclusions, sensitivity analysis was performed after their exclusion, which further showed that the primary findings—the nonlinear association and threshold effect between first-trimester PAC and HDP risk—remained materially unchanged.

### 4.2 Novelty in context

In contrast to the linear models typically applied in prior research, our study employed RCS and piecewise regression analyses, revealing a nonlinear, threshold-dependent relationship between PAC and HDP risk, with an inflection point at approximately 39.30 ng/dL.

This methodological approach may help explain discrepancies across earlier reports. For example, whereas Krzysztof et al. (17) and Robin et al. (18) reported a significant association, Purut et al. (19) and Birukov et al. (20), found no significant relationship. Our results suggest that differences in the distribution of PAC across study populations may contribute to these divergent conclusions. These findings underscore the importance of evaluating nonlinear and conditional associations in future investigations of PAC and HDP.

### 4.3 Potential mechanistic insights and clinical implications

The threshold effect identified in this study suggests a potential hypothesis regarding.

aldosterone’s role in early pregnancy. When first-trimester PAC falls below the threshold of approximately 39.30 ng/dL, aldosterone may support physiological adaptation to early gestation. This interpretation is supported by experimental studies demonstrating that aldosterone can influence placental development through modulation of trophoblast function and regulation of placental factor expression (21–24). The present epidemiological observations are consistent with prior experimental work reporting that adequate aldosterone levels are associated with favorable pregnancy outcomes (21). From a pathophysiological perspective, sustained low first-trimester PAC may relate to inadequate placental implantation and impaired spiral artery remodeling. These disturbances could lead to insufficient uteroplacental perfusion, a process whose dysregulation is a hallmark of HDP. It should be noted that these interpretations are derived from observational data and referenced literature; the underlying biological mechanisms and any causal relationships remain to be established. Conversely, PAC levels at or above 39.30 ng/dL exhibited a positive, but not statistically significant association with HDP risk. This observation may stem from the skewed distribution of PAC values and the limited sample size in the upper concentration stratum of the present cohort. Should future largescale studies establish a significant positive association in this range, surpassing this threshold could be implicated in pathological pathways leading to HDP. Mechanistically, elevated aldosterone is known to enhance mineralocorticoid receptor activity, promote oxidative stress, impair endothelial function, and increase vascular sensitivity to angiotensin II (25, 26). If confirmed, these mechanisms could underpin the potential pathological role of PAC levels exceeding the 39.30 ng/dL threshold in HDP development. It must be emphasized that these mechanistic inferences are drawn from prior literature and remain speculative in relation to the specific threshold identified in this study, their direct relevance and causal role in the observed association require further experimental validation.

These findings carry notable clinical implications. First, the observed association between first-trimester PAC and HDP risk provides epidemiological support for the concept that the pathophysiology of HDP may originate in early gestation. Second, first-trimester PAC emerges as a potential quantifiable biomarker for early HDP risk stratification, which could help identify high−risk individuals, especially those with low PAC levels, who may benefit from intensified surveillance. Third, these observations underscore the importance of shifting preventive strategies toward the first-trimester, allowing earlier and more individualized risk assessment in perinatal care.

### 4.4 Limitations and future directions

Several important limitations should be considered. First, the reported inflection point (39.30 ng/dL) is exploratory, as it was derived and evaluated within the same cohort. The absence of an independent external validation increases the risk of overfitting. Second, the use of a composite HDP outcome, necessitated by limited subtype-specific sample sizes, precluded an assessment of potential differences in the PAC-HDP association across distinct HDP subtypes. Finally, minimal missing data were handled via mean imputation, a practical method that may not fully account for underlying data variability. Future research should focus on three key directions. First, the generalizability and potential clinical utility of the reported PAC threshold must be established through external validation in large, independent, and ethnically diverse cohorts. Second, adequately powered studies with pre-specified designs are needed to determine whether the observed nonlinear association is consistent or varies across specific HDP subtypes. Finally, experimental studies are required to investigate the underlying biological mechanisms and plausibility of the observed nonlinear relationship and threshold.

## 5 Conclusion

First-trimester PAC exhibited a nonlinear association with HDP risk, with an inflection point at 39.30 ng/dL. Below this threshold, higher PAC was significantly associated with a lower risk of HDP. At or above this level, the association was positive but not statistically significant.

## Data Availability

The original contributions presented in the study are included in the article/**Supplementary Material**. Further inquiries can be directed to the corresponding author.

## Supplementary material

Supplementary Table 1 Univariate logistic regression analysis of factors associated with HDP.

Supplementary Table 2 Multivariable logistic regression analysis of independent factors associated with HDP.

Supplementary Table 3 Comparison of baseline characteristics and the prevalence of HDP according to PAC quartiles.

Supplementary Table 4 Association between winsorized PAC and HDP risk.

Supplementary Table 5 Association between PAC and HDP risk after adjustment for recruiting center.

Supplementary Table 6 Association between winsorized PAC and HDP risk after adjustment for recruiting center.

Supplementary Table 7 Threshold effect analysis of winsorized PAC on HDP after adjustment for recruiting center.

Supplementary Table 8 Complete-case analysis of the association between PAC and HDP risk.

Supplementary Table 9 Complete-case analysis of the association between winsorized PAC and HDP risk.

Supplementary Table 10 Threshold effect analysis of winsorized PAC on HDP in complete-case analysis.

Supplementary Table 11 Association between PAC and HDP risk before and after excluding chronic hypertension.

Supplementary Table 12 Association between winsorized PAC and HDP risk before and after excluding chronic hypertension.

Supplementary Table 13 Threshold effect analysis of winsorized PAC on HDP after excluding chronic hypertension.

Supplementary Table 14 Association between PAC and HDP risk before and after adjustment for baseline blood pressure.

Supplementary Table 15 Association between winsorized PAC and HDP risk before and after adjustment for baseline blood pressure.

Supplementary Table 16 Threshold effect analysis of winsorized PAC on HDP before adjustment for baseline blood pressure.

Supplementary Table 17 Association between PAC and HDP risk stratified by plasma renin concentration.

Supplementary Table 18 Threshold effect analysis of winsorized PAC on HDP after adjustment for plasma renin concentration.

Supplementary Table 19 Association between PAC and HDP risk after bootstrap internal validation.

Supplementary Table 20 Threshold effect analysis of winsorized PAC on HDP after bootstrap internal validation.

Supplementary Figure 1 Nonlinear association between winsorized PAC and HDP risk after adjusting for recruiting center. RCS analysis depicting the dose-response relationship between winsorized PAC (x-axis) and the adjusted OR (y-axis) for HDP. A significant nonlinear association was observed (*P* for nonlinearity = 0.009; *P* for overall association = 0.003). The inflection point was identified at 40.20 ng/dL. The model was adjusted for maternal age, BMI, history of HDP, gestational age, baseline SBP, baseline DBP, and recruiting center. BMI, Body mass index; DBP, Diastolic blood pressure; HDP, Hypertensive disorders of pregnancy; PAC, Plasma aldosterone concentration; RCS, Restricted cubic splines; SBP, Systolic blood pressure; OR, Odds Ratio.

Supplementary Figure 2 Nonlinear association between winsorized PAC and HDP risk in complete-case analysis. RCS analysis depicting the dose-response relationship between winsorized PAC (x-axis) and the adjusted OR (y-axis) for HDP. A significant nonlinear association was observed (*P* for nonlinearity = 0.007; *P* for overall association = 0.001). The inflection point was identified at 37.50 ng/dL. The model was adjusted for maternal age, BMI, history of HDP, gestational age, baseline SBP, and baseline DBP. BMI, Body mass index; DBP, Diastolic blood pressure; HDP, Hypertensive disorders of pregnancy; PAC, Plasma aldosterone concentration; RCS, Restricted cubic splines; SBP, Systolic blood pressure; OR, Odds Ratio.

Supplementary Figure 3 Nonlinear association between winsorized PAC and HDP risk after excluding participants with chronic hypertension. RCS analysis depicting the dose-response relationship between winsorized PAC (x-axis) and the adjusted OR (y-axis) for HDP. A significant nonlinear association was observed (*P* for nonlinearity = 0.004; *P* for overall association = 0.002). The inflection point was identified at 39.00 ng/dL. The model was adjusted for maternal age, BMI, history of HDP, gestational age, baseline SBP, and baseline DBP. BMI, Body mass index; DBP, Diastolic blood pressure; HDP, Hypertensive disorders of pregnancy; PAC, Plasma aldosterone concentration; RCS, Restricted cubic splines; SBP, Systolic blood pressure; OR, Odds Ratio.

Supplementary Figure 4 Nonlinear association between winsorized PAC and HDP risk without adjustment for baseline blood pressure. RCS analysis depicting the dose-response relationship between winsorized PAC (x-axis) and the adjusted OR (y-axis) for HDP. A significant nonlinear association was observed (*P* for nonlinearity = 0.005; *P* for overall association = 0.003). The inflection point was identified at 39.00 ng/dL. The model was adjusted for maternal age, BMI, history of HDP, and gestational age. BMI, Body mass index; HDP, Hypertensive disorders of pregnancy; PAC, Plasma aldosterone concentration; RCS, Restricted cubic splines; OR, Odds Ratio.

Supplementary Figure 5 Nonlinear association between winsorized PAC and HDP risk after adjusting for plasma renin concentration. RCS analysis depicting the dose-response relationship between winsorized PAC (x-axis) and the adjusted OR (y-axis) for HDP. A significant nonlinear association was observed (*P* for nonlinearity = 0.006; *P* for overall association = 0.004). The inflection point was identified at 39.30 ng/dL. The model was adjusted for maternal age, BMI, history of HDP, gestational age, baseline SBP, baseline DBP, and renin. BMI, Body mass index; DBP, Diastolic blood pressure; HDP, Hypertensive disorders of pregnancy; PAC, Plasma aldosterone concentration; RCS, Restricted cubic splines; SBP, Systolic blood pressure; OR, Odds Ratio.

Supplementary Figure 6 Nonlinear association between winsorized PAC and HDP risk after bootstrap internal validation. RCS analysis depicting the dose-response relationship between winsorized PAC (x-axis) and the adjusted OR (y-axis) for HDP. A significant nonlinear association was observed (*P* for nonlinearity = 0.006; *P* for overall association = 0.004). The inflection point was identified at 38.20 ng/dL. The model was adjusted for maternal age, BMI, history of HDP, gestational age, baseline SBP, baseline DBP. BMI, Body mass index; DBP, Diastolic blood pressure; HDP, Hypertensive disorders of pregnancy; PAC, Plasma aldosterone concentration; RCS, Restricted cubic splines; SBP, Systolic blood pressure; OR, Odds Ratio.
