# Supplementary material for: Nonlinear association between first-trimester plasma aldosterone concentration and risk of hypertensive disorders of pregnancy: a multicenter prospective cohort study
Source: Front Endocrinol (Lausanne). 2026 May 25;17:1836569. doi: 10.3389/fendo.2026.1836569 (PMC13243126; doi:10.3389/fendo.2026.1836569)
Supplement: Supplementary Table 1 — Univariate logistic regression analysis of factors associated with HDP. [file DataSheet1.docx]

**2. Materials and methods**

**2.1.2 Participant follow-up**

**Follow-up schedule and content:** (1) Antenatal visits (ongoing): At each visit, seated blood pressure was measured according to the baseline protocol, and pregnancy records were reviewed to update information on concurrent complications, and face-to-face interviews were performed to confirm continued participation. (2) Third trimester (32–36 weeks): Follow-up was conducted primarily via outpatient visits. Blood pressure was re-measured, and symptoms suggestive of HDP (e.g., headache, visual disturbances, epigastric pain, significant edema) and medication use were documented. (3) Delivery and postpartum (within 12 weeks): Delivery data were obtained from hospital records. Postpartum telephone interviews assessed blood pressure recovery and complications.

**Loss to follow-up:** Loss to follow-up was defined as failure to collect prespecified data after baseline due to: (1) Unreachable after multiple contact attempts; (2) Voluntary withdrawal; (3) Transfer to a non-collaborating center; or (4) Severe comorbidity leading to withdrawal.

**Minimization and handling:** The sample size calculation accounted for a 10–15% attrition rate. Collaborating hospitals assisted in tracing participants to minimize loss, with a target attrition rate below 20%. For all participants lost to follow-up, the date and reason were documented, and all available data were retained.

**2.6. Quality control**

A comprehensive quality control system was implemented throughout the study to ensure scientific rigor and data reliability.

**2.6.1 Personnel training**

Prior to study initiation, all personnel (obstetricians, nurses, laboratory technicians, and follow-up staff) underwent a standardized training program. Training consisted of two online sessions and one on-site session, covering the study protocol, standardized data collection, blood pressure measurement, sample handling, laboratory procedures, and endpoint adjudication. Certification required passing a practical assessment.

**2.6.2 Quality supervision system**

**(1) Center-based self-inspection:** The head obstetrician at each center was responsible for overseeing all local quality control activities, including preparation, process monitoring, and issue resolution.

**(2) Regional specialized supervision:** The Hypertension Institute of the People’s Hospital of Xinjiang Uygur Autonomous Region acted as the central coordinating body, conducting regular on-site audits, providing technical support, and performing final quality evaluations.

**2.6.3 Process quality control**

**(1) Data collection:** A double-check procedure was implemented. A quality control officer reviewed each data form on the day of completion for accuracy and logic. Corrections were made immediately, and verified data were entered into the electronic database within 24 hours. Additionally, 10% of participants were randomly selected weekly for telephone verification (allowable error rate < 5%).

**(2) Sample management and testing:** All consumables (e.g., EDTA tubes) were centrally provided by the Hypertension Institute to standardize pre-analytical conditions. After recruitment, all plasma samples were analyzed in a single batch at the laboratory of the Hypertension Institute using calibrated instruments and standardized reagents.

**(3) Follow-up and endpoint adjudication**: A standardized follow-up recording system was used. All potential HDP endpoints were centrally re-adjudicated by specialists at the Hypertension Institute, who were blinded to exposure data, by referring to original medical records to ensure diagnostic accuracy.

**2.6.4 Data management**

Data were entered independently by two nurses using a double-entry method. The two datasets were compared, with a target discrepancy rate of < 1%. Any inconsistencies were resolved by reviewing original paper forms, electronic medical records, or via telephone follow-up. The final database was reviewed and locked by an independent statistician prior to analysis.

**Table S1. Univariate logistic regression analysis of factors associated with HDP**

| **Characteristics** | **OR** | **95%CI** | ***P*-value** | **VIF** |
| --- | --- | --- | --- | --- |
| Age, years | 1.053 | 1.014, 1.092 | 0.007 | 1.389 |
| BMI, Kg/m^2^ | 1.107 | 1.069, 1.147 | <0.001 | 1.194 |
| Gravidity | 1.108 | 0.969, 1.268 | 0.135 | 3.400 |
| Parity | 1.243 | 0.986, 1.567 | 0.066 | 2.608 |
| Gestational age, weeks | 1.074 | 0.997, 1.157 | 0.061 | 1.112 |
| SBP, mmHg | 1.075 | 1.056, 1.094 | <0.001 | 1.392 |
| DBP, mmHg | 1.101 | 1.077, 1.125 | <0.001 | 1.308 |
| Miscarriage history, n (%) | 0.932 | 0.614, 1.414 | 0.739 | 1.713 |
| Stillbirth History, n (%) | 1.028 | 0.129, 8.170 | 0.979 | 1.060 |
| HDP history, n (%) | 9.364 | 1.309, 66.978 | 0.026 | 1.061 |
| Diabetes, n (%) | 1.670 | 1.034, 2.698 | 0.036 | 1.061 |
| Hypertension for parents |  |  |  |  |
| father, n (%) | 1.291 | 0.731, 2.281 | 0.379 | 1.160 |
| mother, n (%) | 1.402 | 0.805, 2.442 | 0.233 | 1.114 |
| Leukocyte count × 10^9^/L | 1.037 | 0.947, 1.136 | 0.428 | 1.172 |
| Hemoglobin, g/L | 1.000 | 0.986, 1.015 | 0.968 | 1.152 |
| Platelet count ×10^9^/L | 1.003 | 0.999, 1.006 | 0.110 | 1.114 |
| Fasting glucose, mmol/L | 1.274 | 0.857, 1.896 | 0.232 | 1.098 |
| ALT, U/L | 1.012 | 0.998, 1.026 | 0.096 | 1.764 |
| AST, U/L | 1.022 | 0.999, 1.046 | 0.062 | 1.763 |
| Serum creatinine, μmol/L | 1.015 | 0.998, 1.032 | 0.086 | 1.104 |
| Blood urea nitrogen, mmol/L | 0.985 | 0.836, 1.160 | 0.857 | 1.155 |
| Albumin, g/L | 0.966 | 0.925, 1.008 | 0.113 | 1.127 |
| Calcium levels, mmol/L | 0.728 | 0.326, 1.622 | 0.437 | 1.049 |
| Aldosterone, ng/dL | 0.986 | 0.974, 0.997 | 0.014 | 1.133 |
| Renin, pg/mL | 0.999 | 0.998, 1.001 | 0.429 | 1.191 |

Candidate variables were selected on the basis of *P* < 0.1 and VIF < 5. ALT: Alanine aminotransferase; AST: Aspartate aminotransferase; BMI: Body mass index; Cl: Confidence Interval; DBP: Diastolic blood pressure; HDP: Hypertensive disorders of pregnancy; OR: Odds Ratio; SBP: Systolic blood pressure; VIF: Variance inflation factor.

**Table S2. Multivariable logistic regression analysis of independent factors associated with HDP**

| **Characteristics** | **OR** | **95%CI** | ***P*-value** |
| --- | --- | --- | --- |
| Aldosterone, ng/dL | 0.987 | 0.975, 0.999 | 0.039 |
| BMI, Kg/m2 | 1.056 | 1.015, 1.099 | 0.006 |
| Gestational age, weeks | 1.098 | 1.012, 1.192 | 0.024 |
| SBP, mmHg | 1.052 | 1.031, 1.074 | <0.001 |
| DBP, mmHg | 1.058 | 1.033, 1.085 | <0.001 |
| HDP history, n (%) | 5.074 | 0.597, 43.156 | 0.137 |

Using a stepwise backward procedure (*P* < 0.050 to retain), a final model was derived with age into it on strong clinical grounds. BMI: Body mass index; Cl: Confidence Interval; DBP: Diastolic blood pressure; OR: Odds Ratio; SBP: Systolic blood pressure.

**Table S3. Comparison of baseline characteristics and the prevalence of HDP according to  PAC quartiles**

| **Characteristics** | **PAC, ng/dL** | | | | **H/χ^2^** | ***P*-value** |
| --- | --- | --- | --- | --- | --- | --- |
|  | **˂ 15.59,**  **N = 371** | **15.59-22.31,**  **N = 371** | **22.31-33.40,**  **N = 373** | **≥ 33.40,**  **N = 371** |  |  |
| Age, years | 29.00  (27.00, 34.00) | 29.00  (26.00, 33.00) | 29.00  (27.00, 32.00) | 29.00  (26.00, 31.00) | 10.103 | 0.018^*^ |
| BMI, Kg/m^2^ | 22.77  (20.76, 25.39) | 23.23  (20.70, 26.17) | 22.58  (20.42, 25.37) | 22.43  (19.95, 24.83) | 8.449 | 0.038^*^ |
| Primiparas, n (%) | 163 (43.9%) | 176 (47.4%) | 204 (54.7%) | 199 (53.6%) | 11.678 | 0.009 |
| Gestational age, weeks | 8.00  (7.00, 11.00) | 8.00  (6.00, 11.00) | 8.00  (6.00, 11.00) | 8.00  (6.00, 10.00) | 4.688 | 0.196^*^ |
| SBP, mmHg | 105.00  (100.00, 111.00) | 106.00  (100.00, 113.00) | 105.00  (100.00, 114.00) | 108.00  (100.00, 117.50) | 7.731 | 0.052^*^ |
| DBP, mmHg | 69.00  (63.00, 72.00) | 68.00  (63.00, 71.00) | 66.00  (63.00, 71.00) | 65.00  (63.00, 70.00) | 10.152 | 0.017^*^ |
| Miscarriage history, n (%) | 78 (21.0%) | 87 (23.5%) | 82 (22.0%) | 87 (23.5%) | 0.905 | 0.824^†^ |
| Stillbirth History, n (%) | 1 (0.3%) | 2 (0.5%) | 5 (1.3%) | 2 (0.5%) |  | 0.428^‡^ |
| HDP history, n (%) | 0 (0.0%) | 2 (0.5%) | 1 (0.3%) | 1 (0.3%) |  | 0.764^‡^ |
| Diabetes, n (%) | 1 (0.3%) | 3 (0.8%) | 2 (0.5%) | 3 (0.8%) |  | 0.767^‡^ |
| Hypertension for parents |  |  |  |  |  |  |
| father, n (%) | 28 (7.5%) | 25 (6.7%) | 37 (9.9%) | 35 (9.4%) | 3.312 | 0.346^†^ |
| mother, n (%) | 27 (7.3%) | 34 (9.2%) | 36 (9.7%) | 28 (7.5%) | 1.996 | 0.573^†^ |
| Leukocyte count × 10^9^/L | 7.94  (6.50, 9.10) | 8.14  (6.77, 9.43) | 8.03  (6.56, 9.01) | 7.93  (6.57, 9.35) | 4.941 | 0.176^*^ |
| Hemoglobin, g/L | 123.00  (117.00, 128.00) | 125.00  (120.00, 131.00) | 125.00  (119.50, 131.00) | 127.00  (120.00, 134.00) | 30.814 | <0.001^*^ |
| Platelet Count ×10^9^/L | 245.00  (219.00, 280.00) | 245.00  (219.00, 280.00) | 245.00  (219.00, 281.50) | 245.00  (219.00, 282.00) | 2.192 | 0.534^*^ |
| Fasting glucose, mmol/L | 4.90  (4.60, 5.10) | 4.90  (4.60, 5.11) | 4.90  (4.63, 5.10) | 4.90  (4.65, 5.11) | 5.257 | 0.154^*^ |
| ALT, U/L | 15.00  (12.00, 18.00) | 15.26  (12.00, 19.00) | 14.00  (12.00, 18.00) | 15.00  (11.92, 18.00) | 6.021 | 0.111^*^ |
| AST, U/L | 17.00  (14.00, 19.00) | 17.00  (14.00, 19.00) | 17.00  (14.00, 19.00) | 17.00  (14.00, 18.00) | 1.983 | 0.576^*^ |
| Serum creatinine, μmol/L | 48.00  (43.10, 52.00) | 47.84  (43.00, 52.00) | 47.70  (43.00, 51.00) | 46.00  (42.00, 50.30) | 10.054 | 0.018^*^ |
| Blood urea nitrogen, mmol/L | 3.30  (2.70, 4.40) | 3.30  (2.60, 4.15) | 3.20  (2.60, 3.79) | 3.20  (2.50, 3.90) | 11.407 | 0.010^*^ |
| Albumin, g/L | 44.00  (41.00, 45.50) | 44.38  (41.20, 45.96) | 44.46  (41.70, 47.05) | 44.46  (42.00, 46.90) | 9.473 | 0.024^*^ |
| Calcium levels, mmol/L | 2.33  (2.25, 2.40) | 2.33  (2.25, 2.41) | 2.34  (2.26, 2.42) | 2.34  (2.25, 2.40) | 2.054 | 0.561^*^ |
| Renin, pg/mL | 48.44  (28.43, 91.75) | 83.59  (51.04, 151.76) | 121.02  (77.01, 184.25) | 155.98  (100.39, 247.40) | 306.725 | <0.001^*^ |
| Renin group, n (%) |  |  |  |  | 219.220 | <0.001^†^ |
| ˂ 100.58 | 285 (76.8%) | 215 (58.0%) | 147 (39.4%) | 96 (25.9%) |  |  |
| ≥ 100.58 | 86 (23.2%) | 156 (42.0%) | 226 (60.6%) | 275 (74.1%) |  |  |
| HDP | 49 (13.2%) | 44 (11.9%) | 26 (7.0%) | 26 (7.0%) | 13.352 | 0.004^†^ |

^*^ Kruskal-Wallis rank sum test; ^†^Pearson's Chi-squared test; ^‡^Fisher's exact test.

ALT: Alanine aminotransferase; AST: Aspartate aminotransferase; BMI: Body mass index; DBP: Diastolic blood pressure; HDP: Hypertensive disorders of pregnancy; PAC: plasma aldosterone concentration; SBP: Systolic blood pressure.

**Table S4. Association between winsorized PAC and HDP risk**

| **Characteristics** | **Model 1** | | | **Model 2** | | | **Model 3** | | |
| --- | --- | --- | --- | --- | --- | --- | --- | --- | --- |
|  | **OR** | **95% CI** | ***P*-value** | **OR** | **95% CI** | ***P*-value** | **OR** | **95% CI** | ***P*-value** |
| **PAC** | 0.984 | 0.971, 0.997 | 0.014 | 0.986 | 0.973, 0.999 | 0.040 | 0.986 | 0.972, 0.999 | 0.044 |
| **PAC**^*^ | 0.781 | 0.641, 0.951 | 0.014 | 0.809 | 0.662, 0.990 | 0.040 | 0.805 | 0.652, 0.994 | 0.044 |
| **PAC group** |  |  |  |  |  |  |  |  |  |
| Q1 ˂ 15.59 | Ref. | Ref. |  | Ref. | Ref. |  | Ref. | Ref. |  |
| Q2: 15.59-22.31 | 0.884 | 0.571, 1.366 | 0.579 | 0.811 | 0.517, 1.268 | 0.359 | 0.804 | 0.500, 1.288 | 0.366 |
| Q3: 22.31-33.40 | 0.492 | 0.295, 0.804 | 0.005 | 0.473 | 0.281, 0.781 | 0.004 | 0.478 | 0.278, 0.806 | 0.006 |
| Q4 ≥ 33.40 | 0.495 | 0.297, 0.809 | 0.006 | 0.518 | 0.308, 0.853 | 0.011 | 0.507 | 0.359, 0.857 | 0.012 |
| ***P* for trend** |  |  | < 0.001 |  |  | 0.002 |  |  | 0.002 |

Model 1: no covariates were adjusted.

Model 2: adjusted for age, BMI, and history of HDP.
Model 3: adjusted for age, BMI, history of HDP, gestational age, baseline SBP and DBP.

^*^: standardized PAC.

BMI: Body mass index; CI: Confidence Interval; DBP: Diastolic blood pressure; HDP: Hypertensive disorders of pregnancy; OR: Odds Ratio; PAC: Plasma aldosterone concentration; SBP: Systolic blood pressure.

**Table S5. Association between PAC and HDP risk after adjustment for recruiting center**

| **Characteristics** | **Model 1** | | | **Model 2** | | |
| --- | --- | --- | --- | --- | --- | --- |
|  | **OR** | **95% CI** | ***P*-value** | **OR** | **95% CI** | ***P*-value** |
| **PAC** | 0.987 | 0.974, 0.999 | 0.039 | 0.983 | 0.968, 0.998 | 0.026 |
| **PAC**^*^ | 0.784 | 0.614, 0.975 | 0.039 | 0.722 | 0.542, 0.961 | 0.026 |
| **PAC group** |  |  |  |  |  |  |
| Q1 ˂ 15.59 | Ref. | Ref. |  | Ref. | Ref. |  |
| Q2: 15.59-22.31 | 0.804 | 0.500, 1.288 | 0.366 | 0.863 | 0.535, 1.391 | 0.544 |
| Q3: 22.31-33.40 | 0.478 | 0.278, 0.806 | 0.006 | 0.429 | 0.232, 0.791 | 0.007 |
| Q4 ≥ 33.40 | 0.507 | 0.295, 0.857 | 0.012 | 0.348 | 0.176, 0.692 | 0.003 |

Model 1: adjusted for age, BMI, history of HDP, gestational age, baseline SBP, and baseline DBP.

Model 2: adjusted for age, BMI, history of HDP, gestational age, baseline SBP, baseline DBP, and recruiting center.
^*^: standardized PAC.

BMI: Body mass index; CI: Confidence Interval; DBP: Diastolic blood pressure; HDP: Hypertensive disorders of pregnancy; OR: Odds Ratio; PAC: Plasma aldosterone concentration; SBP: Systolic blood pressure.

**Table S6. Association between winsorized PAC and HDP risk after adjustment for recruiting center**

| **Characteristics** | **Model 1** | | | **Model 2** | | |
| --- | --- | --- | --- | --- | --- | --- |
|  | OR | 95% CI | *P*-value | OR | 95% CI | *P*-value |
| **PAC** | 0.986 | 0.972, 0.999 | 0.044 | 0.981 | 0.964, 0.998 | 0.028 |
| **PAC**^*^ | 0.805 | 0.652, 0.994 | 0.044 | 0.743 | 0.570, 0.969 | 0.028 |
| **PAC group** |  |  |  |  |  |  |
| Q1 ˂ 15.59 | Ref. | Ref. |  | Ref. | Ref. |  |
| Q2: 15.59-22.31 | 0.804 | 0.500, 1.288 | 0.366 | 0.863 | 0.535, 1.391 | 0.544 |
| Q3: 22.31-33.40 | 0.478 | 0.278, 0.806 | 0.006 | 0.429 | 0.232, 0.791 | 0.007 |
| Q4 ≥ 33.40 | 0.507 | 0.359, 0.857 | 0.012 | 0.348 | 0.176, 0.692 | 0.003 |

Model 1: adjusted for age, BMI, history of HDP, gestational age, baseline SBP, and baseline DBP.

Model 2: adjusted for age, BMI, history of HDP, gestational age, baseline SBP, baseline DBP, and recruiting center.

^*^: standardized PAC.

BMI: Body mass index; CI: Confidence Interval; DBP: Diastolic blood pressure; HDP: Hypertensive disorders of pregnancy; OR: Odds Ratio; PAC: Plasma aldosterone concentration; SBP: Systolic blood pressure.

**Table S7. Threshold effect analysis of winsorized PAC on HDP after adjustment for recruiting center**

|  | **OR (95% CI)**^*^ | ***P*-value** |
| --- | --- | --- |
| **Fitting by standard Logistic regression model** | 0.981 (0.964, 0.998) | 0.028 |
| **Fitting by piecewise Logistic regression model (break-points = 40.20 ng/dL)** |  |  |
| PAC < 40.20 | 0.953 (0.926, 0.980) | <0.001 |
| PAC ≥ 40.20 | 1.022 (0.988, 1.058) | 0.209 |
| **Log likelihood ratio** |  | 0.011 |

^*^Adjusted for: age, BMI, history of HDP, gestational age, baseline SBP, baseline DBP, and recruiting center.

BMI: Body mass index; CI: Confidence Interval; DBP: Diastolic blood pressure; HDP: Hypertensive disorders of pregnancy; OR: Odds Ratio; PAC: Plasma aldosterone concentration; SBP: Systolic blood pressure.

**Table S8. Complete-case analysis of the association between PAC and HDP risk**

| **Characteristics** | **Model 1** | | | **Model 2** | | | **Model 3** | | |
| --- | --- | --- | --- | --- | --- | --- | --- | --- | --- |
|  | **OR** | **95% CI** | ***P*-value** | **OR** | **95% CI** | ***P*-value** | **OR** | **95% CI** | ***P*-value** |
| PAC | 0.986 | 0.974, 0.997 | 0.017 | 0.988 | 0.976, 0.999 | 0.045 | 0.987 | 0.974, 0.999 | 0.044 |
| PAC^*^ | 0.770 | 0.613, 0.942 | 0.017 | 0.800 | 0.635, 0.983 | 0.045 | 0.790 | 0.620, 0.982 | 0.044 |
| PAC group |  |  |  |  |  |  |  |  |  |
| Q1 ˂ 15.59 | Ref. | Ref. |  | Ref. | Ref. |  | Ref. | Ref. |  |
| Q2: 15.59-22.31 | 0.881 | 0.570, 1.362 | 0.570 | 0.808 | 0.517, 1.264 | 0.351 | 0.797 | 0.497, 1.278 | 0.346 |
| Q3: 22.31-33.4 | 0.491 | 0.298, 0.808 | 0.005 | 0.471 | 0.283, 0.784 | 0.004 | 0.473 | 0.278, 0.805 | 0.006 |
| Q4 ≥ 33.4 | 0.502 | 0.305, 0.828 | 0.007 | 0.527 | 0.317, 0.876 | 0.013 | 0.513 | 0.301, 0.874 | 0.014 |

Model 1: no covariates were adjusted.

Model 2: adjusted for age, BMI, and history of HDP.
Model 3: adjusted for age, BMI, history of HDP, gestational age, baseline SBP, and baseline DBP.

^*^: standardized PAC.

BMI: Body mass index; CI: Confidence Interval; DBP: Diastolic blood pressure; HDP: Hypertensive disorders of pregnancy; OR: Odds Ratio; PAC: Plasma aldosterone concentration; SBP: Systolic blood pressure.

**Table S9. Complete-case analysis of the association between winsorized PAC and HDP risk**

| **Characteristics** | **Model 1** | | | **Model 2** | | | **Model 3** | | |
| --- | --- | --- | --- | --- | --- | --- | --- | --- | --- |
|  | **OR** | **95% CI** | ***P*-value** | **OR** | **95% CI** | ***P*-value** | **OR** | **95% CI** | ***P*-value** |
| **PAC** | 0.984 | 0.972, 0.997 | 0.017 | 0.987 | 0.973, 0.999 | 0.048 | 0.986 | 0.972, 0.999 | 0.049 |
| **PAC**^*^ | 0.787 | 0.646, 0.957 | 0.017 | 0.816 | 0.662, 0.990 | 0.048 | 0.809 | 0.650, 0.992 | 0.049 |
| **PAC group** |  |  |  |  |  |  |  |  |  |
| Q1 ˂ 15.59 | Ref. | Ref. |  | Ref. | Ref. |  | Ref. | Ref. |  |
| Q2: 15.59-22.31 | 0.881 | 0.569, 1.362 | 0.570 | 0.808 | 0.515, 1.263 | 0.351 | 0.797 | 0.495, 1.276 | 0.346 |
| Q3: 22.31-33.40 | 0.491 | 0.294, 0.802 | 0.005 | 0.471 | 0.279, 0.778 | 0.004 | 0.473 | 0.275, 0.798 | 0.006 |
| Q4 ≥ 33.40 | 0.502 | 0.301, 0.821 | 0.007 | 0.527 | 0.313, 0.869 | 0.013 | 0.513 | 0.298, 0.867 | 0.014 |
| ***P* for trend** |  |  | < 0.001 |  |  | 0.002 |  |  | 0.002 |

Model 1: no covariates were adjusted.

Model 2: adjusted for age, BMI, and history of HDP.
Model 3: adjusted for age, BMI, history of HDP, gestational age, baseline SBP, and baseline DBP.

^*^: standardized PAC.

BMI: Body mass index; CI: Confidence Interval; DBP: Diastolic blood pressure; HDP: Hypertensive disorders of pregnancy; OR: Odds Ratio; PAC: Plasma aldosterone concentration; SBP: Systolic blood pressure.

**Table S10. Threshold effect analysis of winsorized PAC on HDP in complete-case analysis**

|  | **OR (95% CI)**^*^ | ***P*-value** |
| --- | --- | --- |
| **Fitting by standard Logistic regression model** | 0.986 (0.972, 0.999) | 0.049 |
| **Fitting by piecewise Logistic regression model (break-points = 37.50 ng/dL)** |  |  |
| PAC < 37.50 | 0.959 (0.936, 0.983) | <0.001 |
| PAC ≥ 37.50 | 1.026 (0.996, 1.057) | 0.087 |
| **Log likelihood ratio** |  | 0.006 |

^*^Adjusted for: age, BMI, history of HDP, gestational age, baseline SBP, and baseline DBP.

BMI: Body mass index; CI: Confidence Interval; DBP: Diastolic blood pressure; HDP: Hypertensive disorders of pregnancy; OR: Odds Ratio; PAC: Plasma aldosterone concentration; SBP: Systolic blood pressure.

**Table S11. Association between PAC and HDP risk before and after excluding chronic hypertension**

| **Characteristics** | **Model 1** | | | **Model 2** | | |
| --- | --- | --- | --- | --- | --- | --- |
|  | **OR** | **95% CI** | ***P*-value** | **OR** | **95% CI** | ***P*-value** |
| **PAC** | 0.987 | 0.974, 0.999 | 0.039 | 0.987 | 0.974, 0.999 | 0.044 |
| **PAC**^*^ | 0.784 | 0.614, 0.975 | 0.039 | 0.787 | 0.624, 0.994 | 0.044 |
| **PAC group** |  |  |  |  |  |  |
| Q1 ˂ 15.59 | Ref. | Ref. |  | Ref. | Ref. |  |
| Q2: 15.59-22.31 | 0.804 | 0.500, 1.288 | 0.366 | 0.823 | 0.511, 1.324 | 0.422 |
| Q3: 22.31-33.40 | 0.478 | 0.278, 0.806 | 0.006 | 0.484 | 0.283, 0.828 | 0.008 |
| Q4 ≥ 33.40 | 0.507 | 0.295, 0.857 | 0.012 | 0.505 | 0.294, 0.869 | 0.014 |

Model 1: adjusted for age, BMI, history of HDP, gestational age, baseline SBP, and baseline DBP.

Model 2: After excluding participants with chronic hypertension, the analysis was adjusted for age, BMI, history of HDP, gestational age, baseline SBP, and baseline DBP.

^*:^ standardized PAC.

BMI: Body mass index; CI: Confidence Interval; DBP: Diastolic blood pressure; HDP: Hypertensive disorders of pregnancy; OR: Odds Ratio; PAC: Plasma aldosterone concentration; SBP: Systolic blood pressure.

**Table S12. Association between winsorized PAC and HDP risk before and after excluding chronic hypertension**

| **Characteristics** | **Model 1** | | | **Model 2** | | |
| --- | --- | --- | --- | --- | --- | --- |
|  | **OR** | **95% CI** | ***P*-value** | **OR** | **95% CI** | ***P-*value** |
| **PAC** | 0.986 | 0.972, 0.999 | 0.044 | 0.986 | 0.972, 0.999 | 0.046 |
| **PAC^*^** | 0.805 | 0.652, 0.994 | 0.044 | 0.808 | 0.647, 0.992 | 0.046 |
| **PAC group** |  |  |  |  |  |  |
| Q1 ˂ 15.59 | Ref. | Ref. |  | Ref. | Ref. |  |
| Q2: 15.59-22.31 | 0.804 | 0.500, 1.288 | 0.366 | 0.823 | 0.510, 1.323 | 0.422 |
| Q3: 22.31-33.40 | 0.478 | 0.278, 0.806 | 0.006 | 0.484 | 0.279, 0.821 | 0.008 |
| Q4 ≥ 33.40 | 0.507 | 0.359, 0.857 | 0.012 | 0.505 | 0.290, 0.861 | 0.014 |

Model 1: adjusted for age, BMI, history of HDP, gestational age, baseline SBP, and baseline DBP.

Model 2: After excluding participants with chronic hypertension, the analysis was adjusted for age, BMI, history of HDP, gestational age, baseline SBP, and baseline DBP.

^*^: standardized PAC.

BMI: Body mass index; CI: Confidence Interval; DBP: Diastolic blood pressure; HDP: Hypertensive disorders of pregnancy; OR: Odds Ratio; PAC: Plasma aldosterone concentration; SBP: Systolic blood pressure.

**Table S13. Threshold effect analysis of winsorized PAC on HDP after excluding chronic hypertension**

|  | **OR (95% CI)**^*^ | ***P*-value** |
| --- | --- | --- |
| **Fitting by standard Logistic regression model** | 0.986 (0.972, 0.999) | 0.046 |
| **Fitting by piecewise Logistic regression model (break-points = 39.00 ng/dL)** |  |  |
| PAC < 39.00 | 0.960 (0.937, 0.983) | <0.001 |
| PAC ≥ 39.00 | 1.029 (0.997, 1.062) | 0.074 |
| **Log likelihood ratio** |  | 0.006 |

^*^Adjusted for: age, BMI, history of HDP, gestational age, baseline SBP, and baseline DBP.

BMI: Body mass index; CI: Confidence Interval; DBP: Diastolic blood pressure; HDP: Hypertensive disorders of pregnancy; OR: Odds Ratio; PAC: Plasma aldosterone concentration; SBP: Systolic blood pressure.

**Table S14. Association between PAC and HDP risk before and after adjustment for baseline blood pressure**

| **Characteristics** | **Model 1** | | | **Model 2** | | |
| --- | --- | --- | --- | --- | --- | --- |
|  | **OR** | **95% CI** | ***P*-value** | **OR** | **95% CI** | ***P*-value** |
| **PAC** | 0.987 | 0.974, 0.999 | 0.039 | 0.988 | 0.976, 0.999 | 0.048 |
| **PAC**^*^ | 0.784 | 0.614, 0.975 | 0.039 | 0.801 | 0.634, 0.986 | 0.048 |
| **PAC group** |  |  |  |  |  |  |
| Q1 ˂ 15.59 | Ref. | Ref. |  | Ref. | Ref. |  |
| Q2: 15.59-22.31 | 0.804 | 0.500, 1.288 | 0.366 | 0.820 | 0.522, 1.283 | 0.386 |
| Q3: 22.31-33.40 | 0.478 | 0.278, 0.806 | 0.006 | 0.477 | 0.283, 0.788 | 0.004 |
| Q4 ≥ 33.40 | 0.507 | 0.295, 0.857 | 0.012 | 0.533 | 0.317, 0.880 | 0.015 |

Model 1: adjusted for age, BMI, history of HDP, gestational age, baseline SBP, and baseline DBP.

Model 2: adjusted for age, BMI, history of HDP, and gestational age.
^*^: standardized PAC.

BMI: Body mass index; CI: Confidence Interval; DBP: Diastolic blood pressure; HDP: Hypertensive disorders of pregnancy; OR: Odds Ratio; PAC: Plasma aldosterone concentration; SBP: Systolic blood pressure.

**Table S15. Association between winsorized PAC and HDP risk before and after adjustment for baseline blood pressure**

| **Characteristics** | **Model 1** | | | **Model 2** | | |
| --- | --- | --- | --- | --- | --- | --- |
|  | **OR** | **95% CI** | ***P*-value** | **OR** | **95% CI** | ***P*-value** |
| PAC | 0.986 | 0.972, 0.999 | 0.044 | 0.987 | 0.974, 0.999 | 0.049 |
| PAC^*^ | 0.805 | 0.652, 0.994 | 0.044 | 0.819 | 0.669, 0.999 | 0.049 |
| PAC group |  |  |  |  |  |  |
| Q1 ˂ 15.59 | Ref. | Ref. |  | Ref. | Ref. |  |
| Q2: 15.59-22.31 | 0.804 | 0.500, 1.288 | 0.366 | 0.820 | 0.522, 1.283 | 0.386 |
| Q3: 22.31-33.4 | 0.478 | 0.278, 0.806 | 0.006 | 0.477 | 0.283, 0.788 | 0.004 |
| Q4 ≥ 33.4 | 0.507 | 0.359, 0.857 | 0.012 | 0.533 | 0.317, 0.880 | 0.015 |

Model 1: adjusted for age, BMI, history of HDP, gestational age, baseline SBP, and baseline DBP.

Model 2: adjusted for age, BMI, history of HDP, and gestational age.

^*^: standardized PAC.

BMI: Body mass index; CI: Confidence Interval; DBP: Diastolic blood pressure; HDP: Hypertensive disorders of pregnancy; OR: Odds Ratio; PAC: Plasma aldosterone concentration; SBP: Systolic blood pressure.

**Table S16. Threshold effect analysis of winsorized PAC on HDP before adjustment for baseline blood pressure**

|  | **OR (95% CI)**^*^ | ***P*-value** |
| --- | --- | --- |
| **Fitting by standard Logistic regression model** | 0.987 (0.974, 0.999) | 0.049 |
| **Fitting by piecewise Logistic regression model (break-points = 39.00 ng/dL)** |  |  |
| PAC < 39.00 | 0.963 (0.941, 0.985) | 0.001 |
| PAC ≥ 39.00 | 1.026 (0.996, 1.058) | 0.087 |
| **Log likelihood ratio** |  | 0.008 |

^*^Adjusted for: age, BMI, history of HDP, and gestational age.

BMI: Body mass index; CI: Confidence Interval; HDP: Hypertensive disorders of pregnancy; OR: Odds Ratio; PAC: Plasma aldosterone concentration.

**Table S17. Association between PAC and HDP risk stratified by plasma renin concentration**

| **Subgroup** | **N** | **OR (95% CI)** | ***P*-value** | ***P* for interaction** |
| --- | --- | --- | --- | --- |
| **Overall** | 1486 | 0.986 (0.973, 0.996) | 0.014 |  |
| **Renin (pg/mL)** |  |  |  |  |
| < 100.58 | 743 | 0.990 (0.970, 1.010) | 0.460 | 0.286 |
| ≥ 100.58 | 743 | 0.980 (0.960, 0.999) | 0.015 |  |

HDP: Hypertensive disorders of pregnancy; PAC: Plasma aldosterone concentration.

**Table S18. Threshold effect analysis of winsorized PAC on HDP after adjustment for plasma renin concentration**

|  | **OR (95% CI)**^*^ | ***P*-value** |
| --- | --- | --- |
| **Fitting by standard Logistic regression model** | **0.987 (0.973, 1.001)** | **0.068** |
| **Fitting by piecewise Logistic regression model (break-points = 39.30 ng/dL)** |  |  |
| PAC < 39.30 | 0.961 (0.938, 0.986) | <0.001 |
| PAC ≥ 39.30 | 1.027 (0.995, 1.060) | 0.098 |
| **Log likelihood ratio** |  | **0.010** |

^*^Adjusted for: age, BMI, history of HDP, gestational age, baseline SBP, baseline DBP, and renin.

BMI: Body mass index; CI: Confidence Interval; DBP: Diastolic blood pressure; HDP: Hypertensive disorders of pregnancy; OR: Odds Ratio; PAC: Plasma aldosterone concentration; SBP: Systolic blood pressure.

**Table S19. Association between PAC and HDP risk after bootstrap internal validation**

| **Characteristics** | **Model 1** | | | **Model 2** | | |
| --- | --- | --- | --- | --- | --- | --- |
|  | **OR** | **95% CI** | ***P*-value** | **OR** | **95% CI** | ***P*-value** |
| PAC, ng/dL | 0.987 | 0.974, 0.999 | 0.039 | 0.987 | 0.970, 0.999 | 0.039 |

Model 1: adjusted for age, BMI, history of HDP, gestational age, baseline SBP, and baseline DBP.

Model 2: adjusted for age, BMI, history of HDP, gestational age, baseline SBP, and baseline DBP after bootstrap internal validation.

BMI: Body mass index; CI: Confidence Interval; DBP: Diastolic blood pressure; HDP: Hypertensive disorders of pregnancy; OR: Odds Ratio; PAC: Plasma aldosterone concentration; SBP: Systolic blood pressure.

**Table S20. Threshold effect analysis of winsorized PAC on HDP after bootstrap internal validation**

|  | **OR (95% CI)^*^** | ***P*-value** |
| --- | --- | --- |
| **Fitting by standard Logistic regression model** | 0.990 (0.970, 0.999) | 0.043 |
| **Fitting by piecewise Logistic regression model (break-points = 38.20 ng/dL)** |  |  |
| PAC < 38.20 | 0.960 (0.942, 0.983) | 0.001 |
| PAC ≥ 38.20 | 1.029 (0.990, 1.062) | 0.104 |
| **Log likelihood ratio** |  | 0.008 |

^*^Adjusted for: age, BMI, history of HDP, gestational age, baseline SBP, and baseline DBP.

BMI: Body mass index; CI: Confidence Interval; DBP: Diastolic blood pressure; HDP: Hypertensive disorders of pregnancy; OR: Odds Ratio; PAC: Plasma aldosterone concentration; SBP: Systolic blood pressure.
