# Supplementary figures and images for: Nonlinear association between first-trimester plasma aldosterone concentration and risk of hypertensive disorders of pregnancy: a multicenter prospective cohort study
Source: Front Endocrinol (Lausanne). 2026 May 25;17:1836569. doi: 10.3389/fendo.2026.1836569 (PMC13243126; doi:10.3389/fendo.2026.1836569)

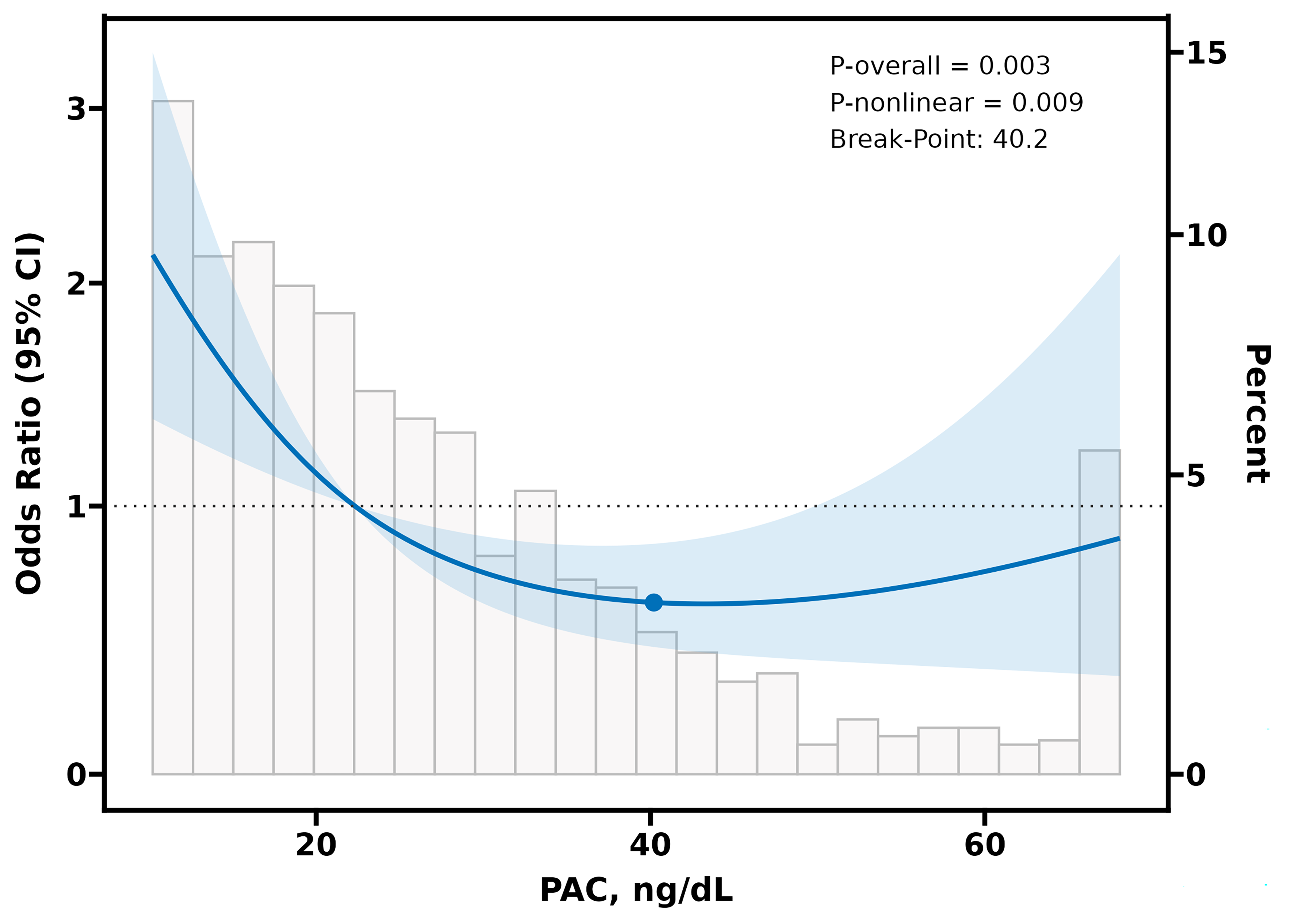

Supplement: Supplementary Figure 1 — Nonlinear association between winsorized PAC and HDP risk after adjusting for recruiting center. RCS analysis depicting the dose-response relationship between winsorized PAC (x-axis) and the adjusted OR (y-axis) for HDP. A significant nonlinear association was observed (P for nonlinearity = 0.009; P for overall association = 0.003). The inflection point was identified at 40.20 ng/dL. The model was adjusted for maternal age, BMI, history of HDP, gestational age, baseline SBP, baseline DBP, and recruiting center. BMI, Body mass index; DBP, Diastolic blood pressure; HDP, Hypertensive disorders of pregnancy; PAC, Plasma aldosterone concentration; RCS, Restricted cubic splines; SBP, Systolic blood pressure; OR, Odds Ratio. [file Image1.tif]

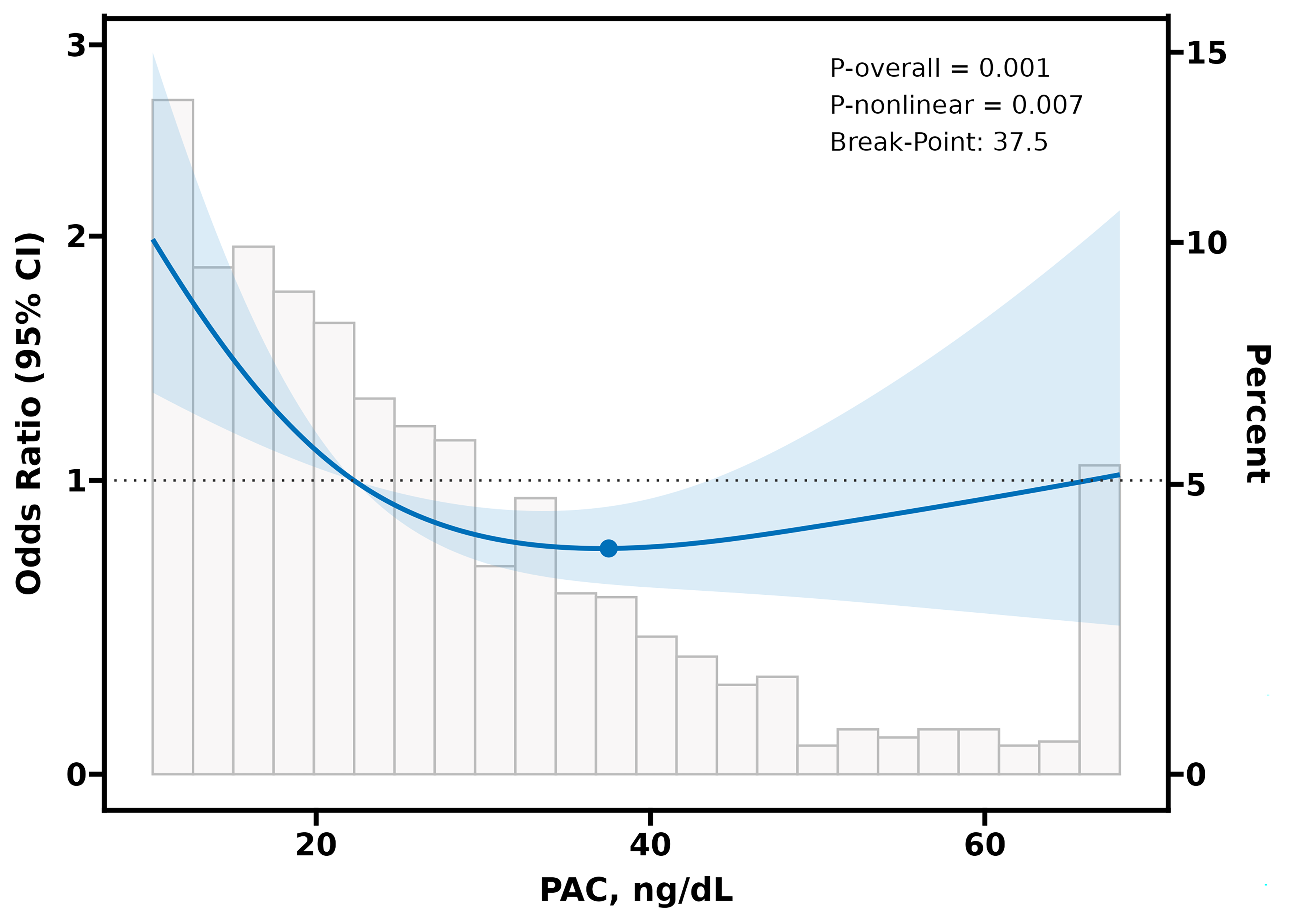

Supplement: Supplementary Figure 2 — Nonlinear association between winsorized PAC and HDP risk in complete-case analysis. RCS analysis depicting the dose-response relationship between winsorized PAC (x-axis) and the adjusted OR (y-axis) for HDP. A significant nonlinear association was observed (P for nonlinearity = 0.007; P for overall association = 0.001). The inflection point was identified at 37.50 ng/dL. The model was adjusted for maternal age, BMI, history of HDP, gestational age, baseline SBP, and baseline DBP. BMI, Body mass index; DBP, Diastolic blood pressure; HDP, Hypertensive disorders of pregnancy; PAC, Plasma aldosterone concentration; RCS, Restricted cubic splines; SBP, Systolic blood pressure; OR, Odds Ratio. [file Image2.tif]

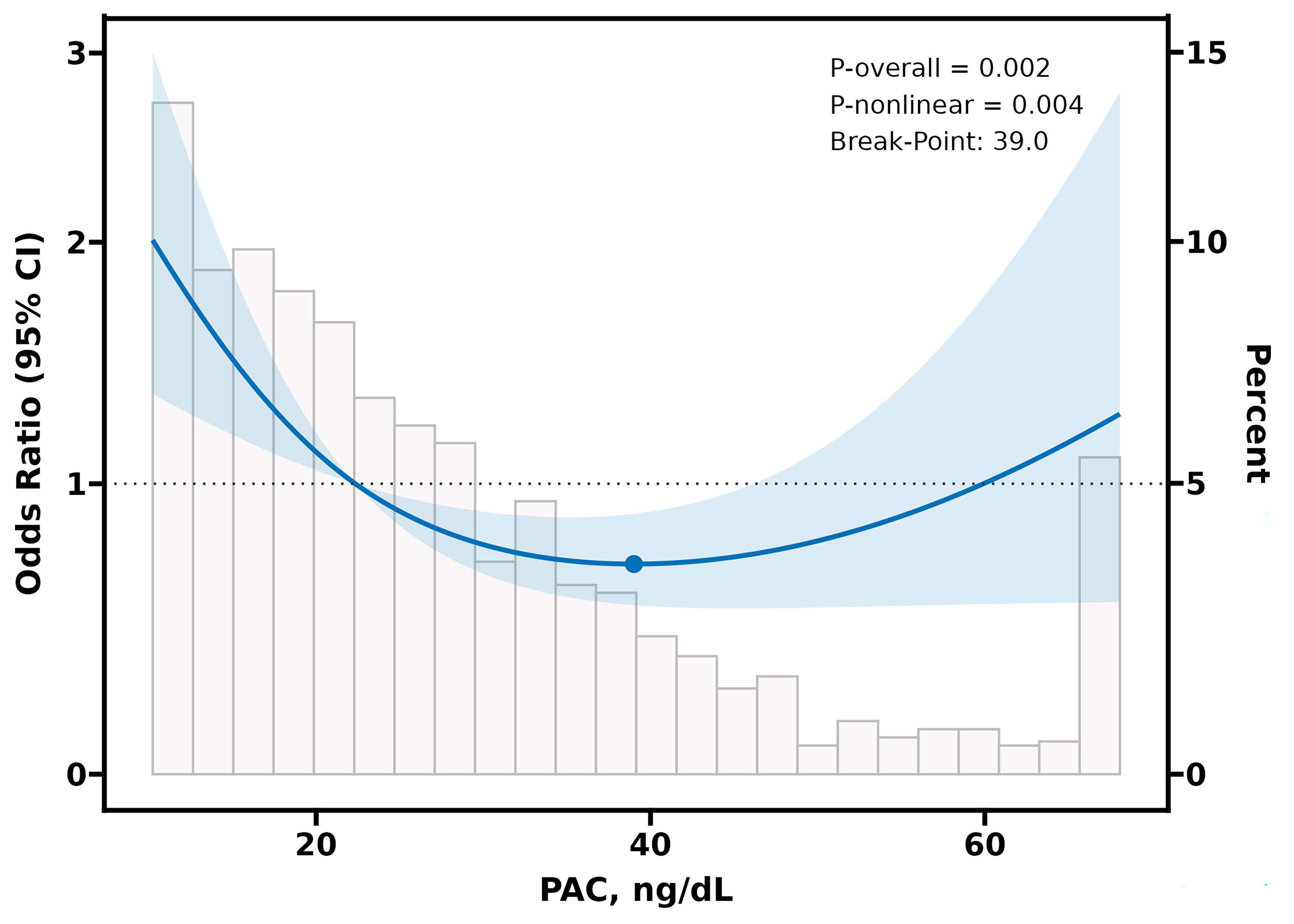

Supplement: Supplementary Figure 3 — Nonlinear association between winsorized PAC and HDP risk after excluding participants with chronic hypertension. RCS analysis depicting the dose-response relationship between winsorized PAC (x-axis) and the adjusted OR (y-axis) for HDP. A significant nonlinear association was observed (P for nonlinearity = 0.004; P for overall association = 0.002). The inflection point was identified at 39.00 ng/dL. The model was adjusted for maternal age, BMI, history of HDP, gestational age, baseline SBP, and baseline DBP. BMI, Body mass index; DBP, Diastolic blood pressure; HDP, Hypertensive disorders of pregnancy; PAC, Plasma aldosterone concentration; RCS, Restricted cubic splines; SBP, Systolic blood pressure; OR, Odds Ratio. [file Image3.tif]

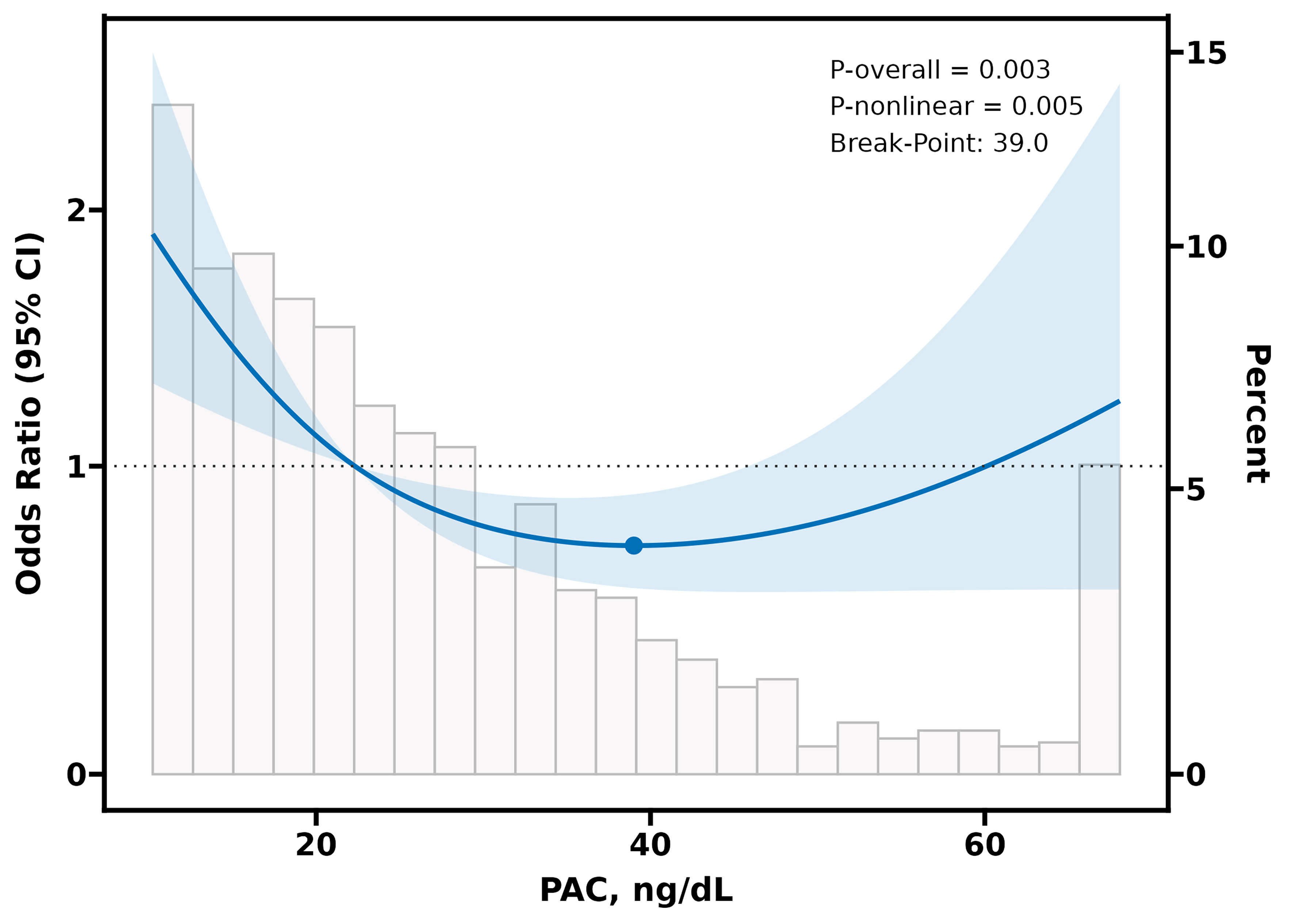

Supplement: Supplementary Figure 4 — Nonlinear association between winsorized PAC and HDP risk without adjustment for baseline blood pressure. RCS analysis depicting the dose-response relationship between winsorized PAC (x-axis) and the adjusted OR (y-axis) for HDP. A significant nonlinear association was observed (P for nonlinearity = 0.005; P for overall association = 0.003). The inflection point was identified at 39.00 ng/dL. The model was adjusted for maternal age, BMI, history of HDP, and gestational age. BMI, Body mass index; HDP, Hypertensive disorders of pregnancy; PAC, Plasma aldosterone concentration; RCS, Restricted cubic splines; OR, Odds Ratio. [file Image4.tif]

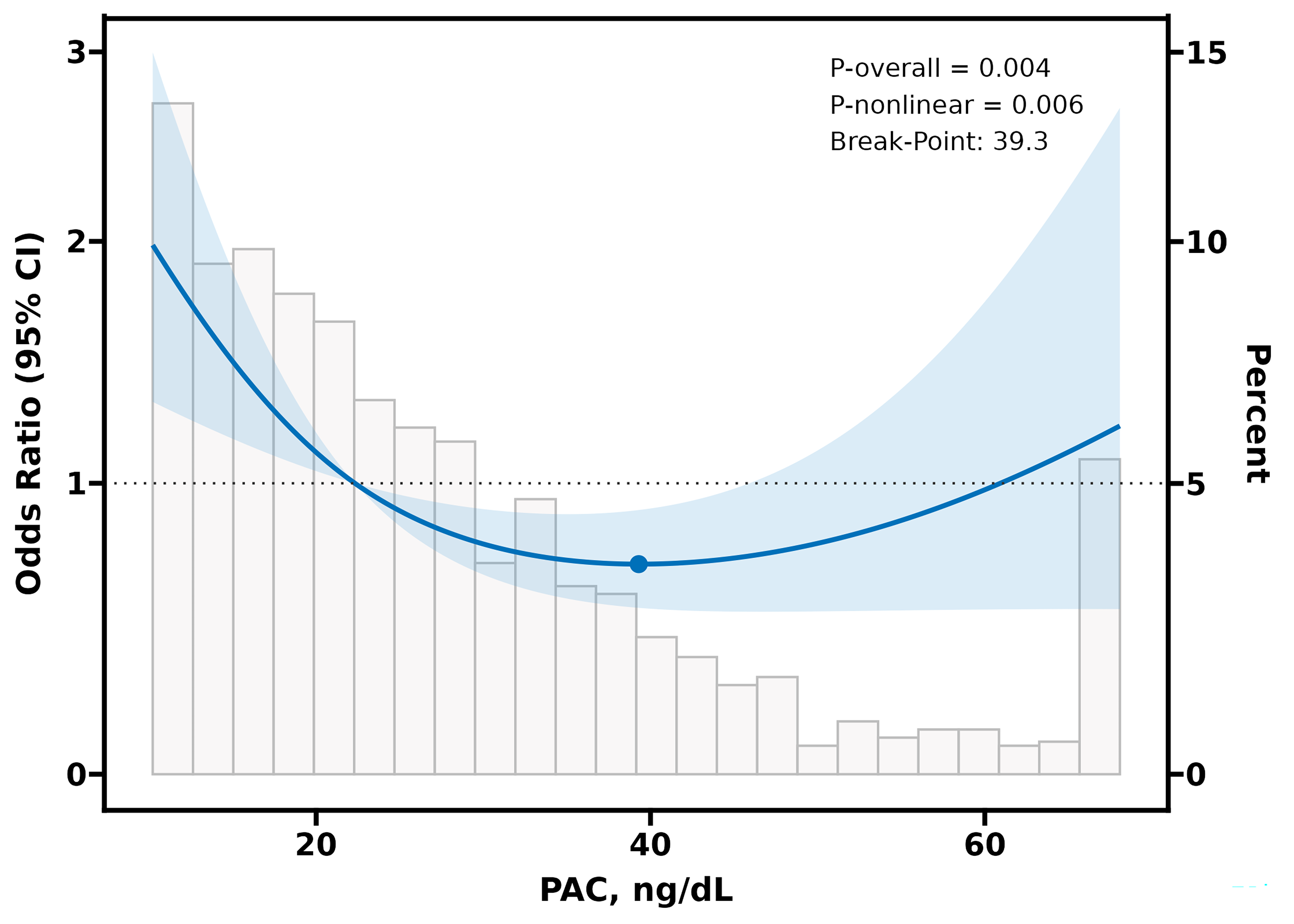

Supplement: Supplementary Figure 5 — Nonlinear association between winsorized PAC and HDP risk after adjusting for plasma renin concentration. RCS analysis depicting the dose-response relationship between winsorized PAC (x-axis) and the adjusted OR (y-axis) for HDP. A significant nonlinear association was observed (P for nonlinearity = 0.006; P for overall association = 0.004). The inflection point was identified at 39.30 ng/dL. The model was adjusted for maternal age, BMI, history of HDP, gestational age, baseline SBP, baseline DBP, and renin. BMI, Body mass index; DBP, Diastolic blood pressure; HDP, Hypertensive disorders of pregnancy; PAC, Plasma aldosterone concentration; RCS, Restricted cubic splines; SBP, Systolic blood pressure; OR, Odds Ratio. [file Image5.tif]

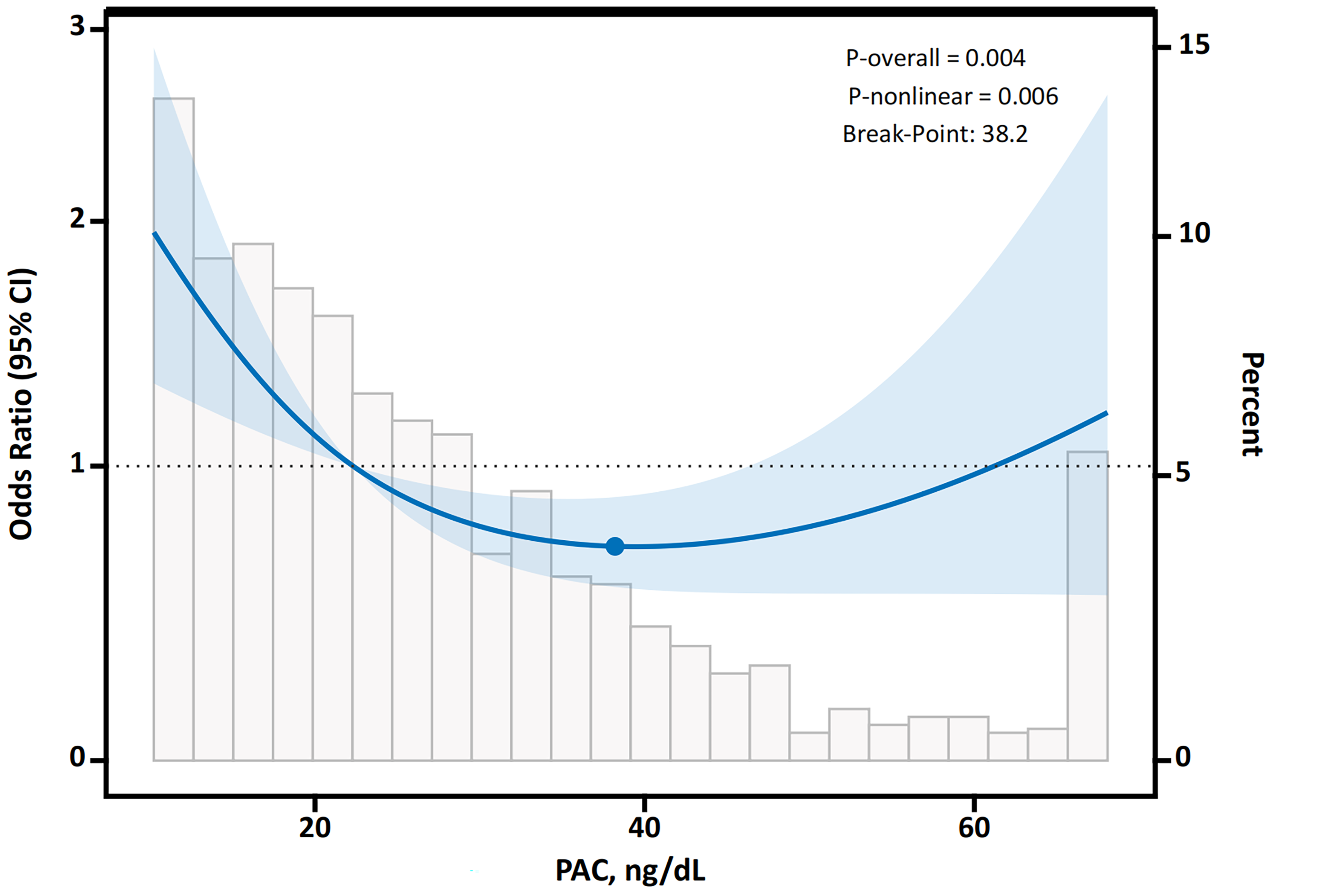

Supplement: Supplementary Figure 6 — Nonlinear association between winsorized PAC and HDP risk after bootstrap internal validation. RCS analysis depicting the dose-response relationship between winsorized PAC (x-axis) and the adjusted OR (y-axis) for HDP. A significant nonlinear association was observed (P for nonlinearity = 0.006; P for overall association = 0.004). The inflection point was identified at 38.20 ng/dL. The model was adjusted for maternal age, BMI, history of HDP, gestational age, baseline SBP, baseline DBP. BMI, Body mass index; DBP, Diastolic blood pressure; HDP, Hypertensive disorders of pregnancy; PAC, Plasma aldosterone concentration; RCS, Restricted cubic splines; SBP, Systolic blood pressure; OR, Odds Ratio. [file Image6.tif]
